# Timescale of environmental change modulates metabolic guild cohesion in microbial communities

**DOI:** 10.1093/ismejo/wraf186

**Published:** 2025-08-22

**Authors:** Kyle Crocker, Abigail Skwara, Rathi Kannan, Arvind Murugan, Seppe Kuehn

**Affiliations:** Department of Ecology and Evolution, The University of Chicago, Chicago, IL 60637, United States; Center for the Physics of Evolving Systems, The University of Chicago, Chicago, IL 60637, United States; Center for Living Systems, The University of Chicago, Chicago, IL 60637, United States; Department of Ecology and Evolutionary Biology, Yale University, New Haven, CT 06520, United States; Center for the Physics of Evolving Systems, The University of Chicago, Chicago, IL 60637, United States; Center for Living Systems, The University of Chicago, Chicago, IL 60637, United States; Pritzker School of Molecular Engineering, The University of Chicago, Chicago, IL 60637, United States; Center for the Physics of Evolving Systems, The University of Chicago, Chicago, IL 60637, United States; Center for Living Systems, The University of Chicago, Chicago, IL 60637, United States; Department of Physics, The University of Chicago, Chicago, IL 60637, United States; National Institute for Theory and Mathematics in Biology, Northwestern University and The University of Chicago, Chicago, IL, 60611, United States; Department of Ecology and Evolution, The University of Chicago, Chicago, IL 60637, United States; Center for the Physics of Evolving Systems, The University of Chicago, Chicago, IL 60637, United States; Center for Living Systems, The University of Chicago, Chicago, IL 60637, United States; National Institute for Theory and Mathematics in Biology, Northwestern University and The University of Chicago, Chicago, IL, 60611, United States

**Keywords:** microbial ecology, metabolic guilds, consumer resource models

## Abstract

Microbial communities experience environmental fluctuations across timescales from rapid changes in moisture, temperature, or light levels to long-term seasonal or climactic variations. Understanding how microbial populations respond to these changes is critical for predicting the impact of perturbations, interventions, and climate change on communities. Because communities typically harbor tens to hundreds of distinct taxa, the response of microbial abundances to perturbations is potentially complex. However, even though taxonomic diversity is high, in many communities taxa can be grouped into metabolic guilds of strains with similar metabolic traits. These guilds effectively reduce the complexity of the system by providing a physiologically motivated coarse-graining. Here, using a combination of simulations, theory, and experiments, we show that the response of guilds to nutrient fluctuations depends on the timescale of those fluctuations. Rapid changes in nutrient levels drive cohesive, positively correlated abundance dynamics within guilds. For slower timescales of environmental variation, members within a guild begin to compete due to similar resource preferences, driving negative correlations in abundances between members of the same guild. Our results provide a route to understanding the relationship between metabolic guilds and community response to changing environments, as well as an experimental approach to discovering metabolic guilds via designed nutrient perturbations to communities.

## Introduction

Natural microbial communities, in contexts ranging from human hosts to soils, are buffeted by perturbations due to changes in host physiology, moisture, nutrients, pH, and temperature. Understanding how these environmental fluctuations impact community composition, interactions [1], and ultimately metabolic processes [2] is of critical importance. For example, changes in moisture, temperature, nutrients, and pH in soils impact the production of greenhouse gasses [3–8]. Understanding the response of these consortia to environmental fluctuations remains a challenge, however, because they harbor hundreds of distinct taxa. In principle, the abundance of each taxon might respond to perturbations via many distinct mechanisms. For example, changes in nutrient availability can impact interactions by relieving competition, whereas changes in moisture and pH can alter oxygen and nutrient availability, respectively [2, 9, 10]. Due to the diversity of taxa and mechanisms that can impact their abundances, one might expect that the response of a community to environmental perturbations should be complex and high-dimensional.

Despite the potential complexity of community responses to perturbations, empirical and theoretical results suggest that communities are comprised of groups of taxa, or metabolic guilds, that perform similar functional roles. For example, in anaerobic digesters groups of taxa specialize in performing distinct steps in the fermentation cascade [11, 12]. Similarly, in marine snow-degrading communities, groups of taxa perform polysaccharide degradation, oligomer uptake, and cross-feeding of excreted metabolites [13]. These metabolic guilds are comprised of multiple coexisting taxa with similar metabolic preferences. As a result, even though communities often retain hundreds of members, in many cases, these taxa can be grouped into metabolic guilds, with members of a guild exhibiting similar metabolic preferences. Metabolic guilds are thought to emerge, at least in part, from trade-offs in microbial traits, e.g. the trade-off between the rate of gluconeogenesis or glycolysis [14], leading to strains specializing in either sugar or acid catabolism [15].

The low number of metabolic guilds relative to the number of strains in a community suggests that it might be much simpler to understand how guilds respond collectively to environmental perturbations, rather than dissecting how each strain responds individually [16]. Because members of a single guild participate in similar metabolic transformations, it might be reasonable to assume guilds respond cohesively to environmental perturbations. Indeed, there is empirical evidence that metabolic guilds respond collectively to changing environmental conditions. For example, complex soil communities in bioreactors exhibit reproducible transitions between denitrification and dissimilatory reduction to ammonia as the carbon-to-nitrogen ratio is varied, reflecting changing dominant metabolic guilds in the system. This transition is thought to arise from the stoichiometric differences between denitrification and DNRA [17]. Similar patterns are observed in large-scale metagenomic surveys of soil microbiomes [1]. Likewise, distinct metabolic guilds in the cow rumen microbiome change in abundance in response to changes in lactate production during fiber fermentation [18]. Theoretically, the notion of metabolic guilds has been described using a modular structure in the traits that members of a community possess [19]. Therefore, metabolic guilds potentially provide a route to understanding how communities collectively respond to environmental change. Specifically, rather than dissect the response of each strain in a system to a perturbation, it might be sufficient to understand how metabolic guilds respond to environmental changes [20, 21]. In this sense, metabolic guilds might enable a more coarse-grained view of the response of communities to environmental change, for instance by describing the community in terms of a few metabolic guilds rather than hundreds or thousands of species [22].

Here, we investigate the idea that metabolic guilds may provide insight into the collective response of microbial communities to environmental change using simulations and experiments. In simulation, we find that the response of metabolic guilds depends on the timescale of environmental changes. Rapid changes in nutrient levels drive correlated dynamics between guild members, with members of the same guild increasing or decreasing their abundances cohesively across the guild. In this fast fluctuation regime, the community-level response reflects the guild structure. In contrast, when environmental fluctuations are slow, abundance dynamics are dominated by intra-guild competition. In this regime, the abundance of members of the same guild exhibit negatively correlated dynamics due to competitive interactions, and the community-level response does not reflect the guild structure. We characterize this behavior quantitatively by showing that the strength of intra-guild cohesion increases as the number of species in the guild decreases, and that the rate of transition from a correlated to an anticorrelated regime is inversely related to the death rate. Next, we turn to synthetic community serial batch culture experiments to test the theoretical prediction that intra-guild abundances are correlated on short timescales and anticorrelated on long timescales. We find that for one experimental guild, abundance correlations indeed decline at longer timescales, consistent with the theoretical expectations. For another guild, however, we see correlations persisting on long timescales. We then modify our theoretical model to show that such behavior is characteristic of cross-feeding interactions, suggesting that intra-guild correlation dynamics provide information about the interaction mechanisms.

## Materials and methods

### Consumer-resource model simulations

Consumer-resource model simulations were performed via the numerical integration of Equations 2, 4, and S31 using the implicit Runge–Kutta method “Radau” implemented in SciPy [23].

#### Random generation of environmental fluctuation rate and phase

To generate environmental fluctuations, we parameterize the resource influx rates


(1) \begin{align*}& K_\alpha(t) = K_{A,\alpha} \sin{\big(\omega_\alpha t - \phi_\alpha \big)} + K_{0,\alpha}\end{align*}

by randomly drawing $\omega _\alpha $ from a uniform distribution from from $\langle \omega \rangle - 0.1 \langle \omega \rangle $ to $\langle \omega \rangle + 0.1 \langle \omega \rangle $, where $\langle \omega \rangle $ is the average fluctuation frequency. The phases $\phi _\alpha $ are drawn from a uniform distribution from $-\pi $ to $\pi $.

#### Random generation of $G$

The growth rate matrix $G$ was generated by first choosing average uptake rates $\langle r \rangle $ and a constant yield $\gamma $. Uptake rate and yield matrices were then constructed such that

(i) Each element had a probability of $p_{f}$ to deviate from the block structure (nonzero uptake rate and yield outside of the blocks, zero uptake rate and yield inside of the blocks).
  (a) By default, $p_{f} = 0.1$, but this value is changed as indicated for the simulations associated with Fig. 3A and B.
(ii) Each nonzero uptake rate is drawn from a uniform distribution from $\langle r \rangle - \Delta G/\gamma $ to $\langle r \rangle + \Delta G/\gamma $, so that the growth rate varies from $\langle g \rangle - \Delta G$ to $\langle g \rangle + \Delta G$.
  (a) By default, $\Delta G = 0.1 \langle r \rangle \gamma $, but this value is changed as indicated for the simulations associated with Figs 3 and S3.

#### Random generation of batch environments

To randomly generate environments for the batch culture simulations (Fig. S4), each initial resource value $K_{B,\alpha }$ was set to either 0 or the default value of 1, each with probability 0.5. This differs from the choice of experimental environments and the corresponding simulation of the experiment (Figs 4 and S5). These are described in the experimental methods section below.

#### Calculation of strain–strain correlation matrix across time

To calculate the strain–strain correlation matrices in continuous simulations (Figs 2 and 3), the following procedure was used. First, strain abundances were initialized at their steady-state values corresponding to constant resource influx at the average values $K_\alpha (t) = K_{0,\alpha }$. This was done to eliminate transient abundance dynamics at the beginning of the simulation. Next, abundance values for each strain were calculated for the fluctuating environment described in Equation 3 with timestep duration equal to the environmental fluctuation timescale $T$ (timestep $\Delta t = T$). These abundance dynamics were then mean-centered for each strain. Finally, an array consisting of 1000 mean-centered abundance timepoints for each strain (corresponding to a total duration of $1000T$) was passed to the NumPy function corrcoef [24] to compute the correlation matrix.

#### Calculation of average $\langle \rho _{g} \rangle $

To calculate average $\langle \rho _{g} \rangle $ (Figs 3 and S3), the strain–strain correlation matrix $\rho _{i,j} = \text{cov}(x_{i},x_{j})/(\sigma _{x_{i}} \sigma _{x_{j}})$ over time was calculated (see the previous section) for either 10 ($\langle g \rangle $ and $\Delta G$) or 6 ($K_{A,\alpha } / K^{0}_{A,\alpha }$) randomly generated $G$ matrices with randomly generated fluctuation rates $\omega _\alpha $ for each set of parameters. For each simulation, the average correlation was calculated across all pairs of strains ($N_{g}$ total pairs) in each guild. The average of these pairs $\langle \rho _{g} \rangle = \frac{\sum _{i \in g} \sum _{j \in g, j <i} \rho _{i,j}}{N_{g}}$ was then averaged across simulations (instantiations of $G$ and $\omega _\alpha $) to compute average $\langle \rho _{g} \rangle $.

#### Calculation of $T_{c}$

To determine the fluctuation rate at which average $\langle \rho _{g} \rangle $ switched sign, $T_{c}$, we fit average $\langle \rho _{g} \rangle $ to a smoothing spline with degree $k=5$ [25]. We then used the spline fit to interpolate between simulated values of average $\langle \rho _{g} \rangle $. $T_{c}$ was then defined as the average period at which the interpolated average $\langle \rho _{g} \rangle $ was $0$. Uncertainty in $T_{c}$ was calculated by resampling simulations with replacement at each fluctuation rate 1000 times, recalculating $T_{c}$, and calculating the standard deviation of this ensemble.

#### Simulation parameter values

Unless otherwise specified, the simulation parameter values are as tabulated (Table 1). In the two-block batch simulation (Fig. S4), $R_{0}=0.01$ to ensure consistency with continuous two-block simulations (Figs 2 and 3), but this value was increased to $R_{0}=0.2$ for the simulations using experimental $G$ matrices to ensure numerical stability (Fig. 4). Previous work shows that the value of this parameter should not meaningfully impact dynamics in the batch case when it is much smaller than the initial resource levels supplied at the beginning of each cycle [26]. This is satisfied in each case, because $K^{b}_\alpha =1$ in the two-block case, and $4<K^{b}_\alpha <13$ in the experimental case.

**Table 1:** Default simulation parameter values.

Parameter	Continuous simulation (Equation 2)	Batch simulation (Equation 4)
$\langle r \rangle $	1	1
$\gamma _{i,\alpha }$	0.2	0.2
$R_{0}$	0.01	0.2
$d_{x}$	0.1	NA
$d_{R}$	0.02	NA
$K_{A,\alpha }$	10	NA
$K_{0,\alpha }$	100	NA
$K_{\alpha }^{b}$	NA	1
$D$	NA	8
$\Delta G$	$0.1 \langle r \rangle \gamma $	$0.1 \langle r \rangle \gamma $
$p_{f}$	$0.1 $	$0.1$

### Experimental methods

#### Characterization of bacterial growth

We characterized growth of 20 bacterial strains by growth in minimal media supplemented with different carbon resources. Each strain was revived from a glycerol stock and grown in 96-deep well plates in 1.2 ml of 0.2X Tryptic Soy Broth (TSB) at 30$^{\circ }$C with shaking for 2 days and was subsequently back-diluted into 0.2X TSB for one more day. Cultures were then washed in carbon-free minimal media and used to inoculate 700} ul of a defined minimal media supplemented with different carbon resources at a low optical density ($\text{OD}_{600}=0.01$) in 48-well optical plates. The minimal media consists of 15} mM ammonium as the assimilatory nitrogen source, 40} mM phosphate buffer with the final medium pH adjusted to 7.3, trace metals and vitamins, and 25 mM of carbon atoms from one of 10 carbon compounds: arabinose, butyrate, deoxyribose, glucuronic acid, glycerol, mannitol, mannose, melibiose, propionate, and raffinose. Minimal media cultures were grown at 30$^{\circ }$C with shaking (500 rpm, Fisherbrand Benchtop Incubating Microplate Shaker) for 72 h with logarithmic sampling to measure optical density ($\text{OD}_{600}$). Growth rates $g_{i,\alpha }$ were inferred via a linear fit to $\log (\text{OD}_{600})$ in exponential phase. Biomass yield per carbon atom was inferred from endpoint $\text{OD}_{600}$ and known input carbon concentration ($\gamma = \Delta \text{OD} / \Delta C$, where $\Delta \text{OD}$ is the change in $\text{OD}_{600}$ during growth cycle and $\Delta C$ is the carbon concentration consumed assuming complete consumption). Finally, uptake rate was calculated using the inferred $g_{i,\alpha }$ and $\gamma _{i,\alpha }$ as follows: $r_{i,\alpha } = g_{i,\alpha } / \gamma _{i,\alpha }$. We emphasize that this calculation is approximate and may be corrupted by incomplete consumption of the carbon source. Growth rates and yields for all strains used for the experiment can be accessed in the code and data repository associated with this manuscript, 10.17605/OSF.IO/J8S2V.

#### Serial-dilution batch culture experiment

The 20 characterized bacterial strains were revived from glycerol stocks as described above and grown to early stationary phase before being used to inoculate 32 media conditions with distinct carbon resource profiles. Each media condition was made with the minimal media described above, amended with a total of 25} mM carbon atoms from a unique combination of one to six carbon resources. Each media condition was inoculated with a uniform mixture of the 20 strains at a starting total $\text{OD}_{600} = 0.01$ in 700 ul in a 48-well optical plate sealed with a breathable film seal. These batch culture plates were grown at 30$^{\circ }$C with shaking (500} rpm, Fisherbrand Benchtop Incubating Microplate Shaker) for 48 h. At the end of 48 h, each batch culture was diluted into fresh media with a 1:10 dilution. The remaining culture was spun down and frozen for downstream DNA extraction.

#### Choice of environments

A total of 32 media conditions, representative of 32 environments, were randomly generated as follows: given a total of 10 carbon resources, each resource had a 25% chance of being selected for a given environment, resulting in environments with different numbers of carbon resources. The total carbon atom concentration in every environment was fixed to 25 mM and evenly partitioned between the resources assigned to that environment. The robustness of the community composition response to this environment selection protocol was assessed using five replicate consumer-resource model simulations of a batch culture instantiated with the 20 strains and measured $G$ used in the experiment (Equation 4). All five replicate simulations demonstrated qualitatively similar responses in the community composition, so a representative set of environments was chosen for the experiment (in particular, the environments corresponding to the first simulation). Simulation results and the set of environments used for the experiment can be accessed in the code and data repository associated with this manuscript, 10.17605/OSF.IO/J8S2V.

#### Sequencing

Genomic DNA was extracted from bacterial cell pellets using the Qiagen DNeasy Blood and Tissue plate-based kit with a modified lysis protocol for Gram-positive bacteria recommended in the kit. To estimate the absolute abundance of bacterial 16S rRNA gene amplicons, we added known quantities of genomic DNA extracted from *Escherichia coli* K-12 (sourced from the Duchossois Family Institute Commensal Isolate Library, Chicago, IL, USA) to the pellet resuspension buffer prior to DNA extraction. 16S rRNA gene amplicon sequencing was performed using the Illumina 16S Metagenomic Sequencing Library Preparation protocol with some modifications. The V3-V4 region was amplified using forward primer 341-b-s-17 with Nextera adapter (TCGTCGGCAGCGTCAGATGTGTATAAGAGACAGCCTACGGGNGGCWGCAG) and reverse primer 806R_Apprill with Nextera adapter (GTCTCGTGGGCTCGGAGATGTGTATAAGAGACAGGGACTACNVGGGTWTCTAAT). Sequences were generated on a MiSeq System (Illumina) using a 2 x 300 bp paired-end v3 reagent kit with a 10% PhiX spike-in.

#### Sequence data processing and ASV-assignment with DADA2

The following preprocessing steps were performed in DADA2 [27]. Raw reads were stripped of primer sequences, truncated after a Phred quality score below 5, and filtered to a maximum expected error of 2 in the forward reads and 5 in the reverse reads based on Phred scores. Amplicon sequence variant (ASV) assignment on processed reads was performed using DADA2 and the recommended analysis pipeline. Forward and reverse reads were denoised separately, then merged and filtered for chimeras. ASV inference was performed by pooling all samples together. After processing, each strain was assigned to one ASV (based on prior 16S rRNA gene assignment by Sanger sequencing performed on axenic cultures of each isolate) and one strain was assigned to two ASVs that were correlated across samples. The sequencing depth was e3-e4 reads per sample. To obtain the absolute abundance of each ASV per sample, the ASV counts in each sample were divided by the spike-in *Escherichia coli* K-12 ASV counts in the same sample.

#### Identification of metabolic guilds

Metabolic guilds were identified by first creating an empirical growth rate matrix ($G$, strain x carbon resource) on a synthetic community, using measured growth rates. Next, this matrix was hierarchically clustered using Ward’s method [28]. Individual guilds were defined using a distance threshold on the resulting dendrogram (95% of the maximum link distance).

#### Calculation of strain–strain correlations across environments $\rho _{i,j,c}$

To calculate the strain–strain correlation matrices at each cycle in batch conditions (Figs 4 and S4), the following procedure was used. First, abundance values for each strain were measured or calculated at the end of each cycle. Second, an array consisting of abundance values for each strain in each environment was passed to the NumPy function corrcoef [24] to compute the correlation matrix for each cycle.

#### Uncertainty calculation of batch intra-guild correlation coefficient $\langle \rho _{g,c} \rangle $

To calculate the uncertainty in $\langle \rho _{g,c} \rangle $ (where brackets indicate averaging over $i,j \in g$, Equation 6; Figs 4C and S4C), environments are first resampled with replacement 1000 times. $\langle \rho _{g,c} \rangle $ is calculated for each resampled set of environments, and the standard deviation of this ensemble is shown by the envelope.

## Results

We analyze a simple mathematical model of communities with metabolic guilds. Studying this model *in silico* and analytically reveals that the timescale of environmental fluctuations determines whether or not members of a guild respond cohesively to these fluctuations.

### Trait-based description of microbial community

Here, we study the response of communities to environmental change using a consumer-resource modeling (CRM) framework. The consumer-resource framework is suitable for interrogating environmental changes because it directly links the environment (resources) and growth. In the CRM framework, environmental fluctuations can be instantiated through time-varying resources.

**Figure 1:**
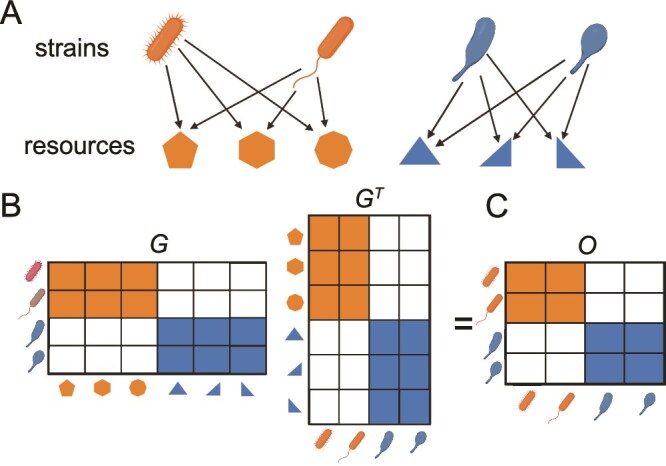
A model for resource consumption preferences and overlap. (A) We study a consumer-resource model, which consists of microbial strains (cartoon microbes) and the resources consumed (shapes). We consider a simple community with two metabolic guilds, represented by the colors. (B) The community description illustrated in (A) is formalized by a “growth matrix” $G$. The rows $i$ of this matrix represent strains, the columns $\alpha $ represent resources, and the entries are the products of uptake rates and yields $r_{i,\alpha } \gamma _{i,\alpha }$ (equivalent to the exponential growth rate in the high resource limit $R_\alpha \gg R_{0}$, Equation 2). (C) The similarity of two strains can be quantified by their “overlap matrix” $O=GG^{T}$.

A CRM framework allows the specification of metabolic guilds by defining groups of taxa with similar metabolic resource preferences. For illustration, consider a four-member community with two metabolic guilds (Fig. 1A, orange and blue) with strains that exclusively consume nonoverlapping nutrients (Fig. 1A, shapes). The strains within each guild compete for shared resources, but do not consume the resources utilized by the strains in the other guild. This block-diagonal guild structure (with blocks of large growth rates along the diagonal corresponding to the resource preference of each guild) is qualitatively consistent with recent empirical work [15, 29].

The guild concept (Fig. 1A) can be formalized by defining a growth matrix, $G$, which specifies the growth rates of each strain on each resource (Fig. 1B). In particular, the rows of this matrix correspond to the growth rates of each strain on each resource $\vec{g_{i}} = \{g_{i,1}, g_{i,2}, g_{i,3},... \}$, where the second index indicates the resource.

This formalism gives rise to a measure of metabolic similarity: the overlap matrix, $O = GG^{T}$ (Fig. 1C). Each element in this matrix $O_{i,j}$ corresponds to the dot product of the growth rate vectors of strains $i$ and $j$, $\vec{g_{i}} \cdot \vec{g_{j}}$. $O$ therefore quantifies the pairwise resource preference overlap of strains in the community, and its structure encodes the guild structure within the community. Groups of strains with high overlap (Fig. 1C, orange and blue blocks) correspond to metabolic guilds of strains with similar resource preferences.

The resource preference matrix ($G$), resource supply rates, and death rates define the dynamics in our consumer-resource model as follows:


(2) \begin{align*}& \begin{aligned} \frac{dx_{i}}{dt} &= x_{i} \left(\displaystyle\sum_{\alpha = 1}^{M} r_{i,\alpha} \gamma_{i,\alpha} \frac{R_\alpha}{R_\alpha + R_{0}} - d_{x}\right)\\ \frac{dR_\alpha}{dt} &= K_\alpha(t) - \displaystyle\sum_{i = 1}^{N} r_{i,\alpha} \frac{R_\alpha}{R_\alpha + R_{0}}x_{i} - R_\alpha d_{R}\\ \end{aligned}\end{align*}

for $N$ strains and $M$ resources, where $x_{i}$ are the consumers, $R_\alpha $ are the resources, $\gamma _{i,\alpha }$ are the yields, $R_{0}$ is the resource affinity, $d_{x}$ is the consumer death rate, $K_\alpha (t)$ are the time-dependent resource influx rates, and $d_{R}$ is the resource depletion rate. Following [26], we have decomposed the growth rates $g_{i,\alpha }$ into the product of a resource uptake rate, $r_{i,\alpha }$, and biomass yield $\gamma _{i,\alpha }$. $r_{i,\alpha }$ has units of resource concentration per unit biomass per unit time and $\gamma _{i,\alpha }$ has units of biomass per unit resource concentration, so $g_{i,\alpha } = r_{i,\alpha } \gamma _{i,\alpha }$ is a rate. In this model, the supply rates of the resources are time-dependent ($K_\alpha (t)$), whereas prior studies treat the supply rate of resources as time-independent and study the steady state of the community at long times [30].

### Abundance dynamics across timescales of environmental fluctuations

**Figure 2:**
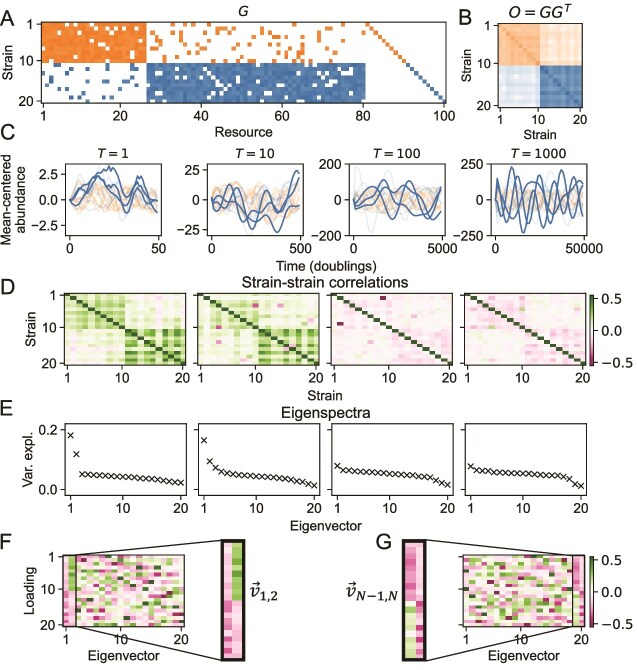
Microbial guilds interacting via resource competition are correlated on short timescales and anticorrelated on long timescales. (A) Matrix ($G$, Fig. 1) enumerating growth rates for strains (rows) on resources (columns). The white entries indicate no growth. The colors indicate growth, with orange and blue demarcating the two guilds and the colormap varying linearly from $0$ to $\text{max}(G)$. $G$ is corrupted by giving $p_{f} = 0.1$ probability for each entry of $G$ to switch between $0$ and $U(\langle r \rangle \gamma - \Delta G,\langle r \rangle \gamma + \Delta G)$, where $U(a,b)$ denotes a uniform distribution on the interval $(a,b)$. $\langle r \rangle $, $\gamma $, and $\Delta G$ are set to default values (Methods). Finally, each strain is given a private resource to ensure coexistence. (B) Overlap matrix $O=GG^{T}$ quantifies the pairwise similarity of strain–strain resource preferences. The colors indicate guilds, and the colormap varies linearly from $0$ to $\text{max}(O)$. (C) Simulated mean-centered abundance dynamics are shown across four timescales. The title gives the timescale $T = 2 \pi / \langle \omega \rangle $ of environmental fluctuations in units of approximate doubling time on a single resource, where $\langle \omega \rangle $ is the average resource fluctuation rate. Abundance dynamics are colored according to guild and dynamics from strains 11, 12, and 17 are highlighted to illustrate the loss of intra-guild cohesion as the timescale increases. Axes scales are changed in each panel so that dynamics are visible. (D) Pearson’s correlation coefficient for the abundance dynamics of each strain pair is shown at each fluctuation timescale $T$ (Methods). Correlation coefficients are calculated over the entire timeseries, which is $1000T$ for each $T$. (E) Rank-ordered variance explained by the eigenvectors of the corresponding correlation matrix in (D). (F) Eigenvectors for $T=1$ doubling case (left panels, (D), (E)), ordered according to the rank of the eigenvalues. The eigenvectors corresponding to the two largest eigenvalues reflect the block structure. In particular, the strains in the blue block are weighted most heavily in $\vec{v}_{1}$, and the strains in the orange block are weighted most heavily in $\vec{v}_{2}$. The corresponding eigenvalues are above the background (E, left panel), indicating a cohesive intra-guild response to fast environmental fluctuations. The colormap is the same as in panel G. (G) Eigenvectors for $T=1000$ doublings, ordered according to the rank of the eigenvalues. The eigenvectors of the two smallest eigenvalues reflect the block structure. In particular, the strains in the blue block are weighted most heavily in $\vec{v}_{N}$, and the strains in the orange block are weighted most heavily in $\vec{v}_{N-1}$. These are the smallest eigenvalues, indicating a there is no cohesive intra-guild response to environmental fluctuation for slow fluctuations.

We simulate the abundance dynamics of a community subject to a range of environmental fluctuation frequencies. We randomly generate a community that has two metabolic guilds by defining a resource preference matrix $G$ (Fig. 2A; Methods). The community includes 20 strains consuming 100 different resources. The two metabolic guilds each comprise 10 strains (Fig. 2A, orange and blue blocks). Noise is added to this matrix, giving rise to members of the orange guild consuming some nutrients that are predominantly utilized by the blue guild and the converse. The role of this noise is explored below. Finally, the $G$ matrix includes a set of private resources, one per strain, that ensures coexistence without biasing the composition within metabolic guilds (Fig. 2A, diagonal block; Supplementary Information). Guilds become clear in the overlap matrix $O$ (Fig. 2B).

The dynamics of the community are governed by Equation 2, with environmental fluctuations taking the form of temporal changes in the input resource vector $\vec{K}(t)$. To introduce environmental fluctuations at different timescales, the input rate of each resource $K_\alpha $ fluctuates as a sinusoid.


(3) \begin{align*}& K_\alpha(t) = K_{A,\alpha} \sin{\big(\omega_\alpha t - \phi_\alpha \big)} + K_{0,\alpha}\end{align*}

For a given simulation, we set a timescale of fluctuations via the frequencies $\omega _\alpha $, which vary by 10% across resources around the average value (Methods). The average resource fluctuation frequency ($\langle \omega \rangle $) defines the timescale of environmental fluctuations, $T = 2 \pi / \langle \omega \rangle $.

Such periodic fluctuations are qualitatively similar to many environmental dynamics experienced by natural communities, such as diel cycles [31], tidal patterns [32], and seasonal variation [33]. Because each resource fluctuates at a slightly different frequency, these fluctuations result in a continuously changing environment with a characteristic timescale set by $\langle \omega \rangle $. To study the response of the system at different timescales, we vary this average frequency.

Abundance dynamics (colored by guild identity) were simulated for environmental fluctuations across a range of timescales (Fig. 2C). We define timescales in terms of the approximate doubling time, $\ln{(2)} / \langle g \rangle $, across all members of the community. Fast fluctuations have a timescale $T$, which has a similar order of magnitude to the approximate doubling time in the community (Fig. 2C, left two panels). Slow environmental fluctuations mean that $T$ is much longer than the approximate doubling time (Fig. 2C, right two panels). Examination of the abundance dynamics (Fig. 2C) suggests that for fast timescales of environmental fluctuations (Fig. 2C, left panels), members of the same guild exhibit cohesive dynamics, with abundances of taxa within the same guild rising and falling together in response to environmental changes. In contrast, when environmental fluctuations are slow, the abundances of members of the same guild do not exhibit cohesive dynamics (Fig. 2C, right panels).

### Metabolic guilds dominate response for fast but not slow environmental fluctuations

To formalize the qualitative observations of the abundance dynamics (Fig. 2C), we computed correlations of abundances across each pair of strains in the community (Fig. 2D; Methods). For fast environmental fluctuations, we observe positively correlated abundance dynamics of members of the same guild (Fig. 2D, green blocks in left panels) and converse for long-timescale fluctuations (Fig. 2D, pink blocks in right panels).

**Fast environmental fluctuations drive correlated, cohesive response:** When environmental fluctuations are fast, the community does not have time to approach a steady state. In this case, the strain abundance dynamics are dominated by the environment defined by the resource influx rates $\vec{K}$. Strain abundances respond to changes in the availability of their preferred resources. This leads to the correlations, $\rho _{i,j} =\text{cov}(x_{i},x_{j})/(\sigma _{x_{i}} \sigma _{x_{j}})$, in abundance dynamics of similar strains (Fig. 2C and D, left panels).

To examine the cohesive response more quantitatively, we performed principal component analysis (PCA) on scaled abundance dynamics. PCA yields eigenvectors and corresponding eigenvalues, where each eigenvector represents a pattern of coordinated response among species, and its associated eigenvalue quantifies the importance of that type of response. In the quickly fluctuating regime (Fig. 2E, left two panels), the first eigenvalue captures the coordinated dynamics of the guild with more resources (blue), whereas the second eigenvalue captures the coordinated dynamics of the guild with fewer resources (orange). This can be seen in the eigenvectors (Fig. 2F), where the first (and largest) eigenvector has high magnitude loadings for strains in the blue guild, whereas the second (and second-largest) eigenvector has high magnitude loadings for strains in the orange guild. This indicates that the highest levels of variation in the abundance dynamics correspond to the coordinated response of the two guilds. Overall, therefore, the response of the community to fast environmental fluctuations matches the structure of the trait matrix, leading to a cohesive response at the guild level.

**Slow environmental fluctuations cause competition to drive noncohesive response:** The environment-dominated regime can be contrasted to the regime defined in the opposite limit—very slow environmental fluctuations. In this regime, the strain abundances equilibrate much more quickly than the resource environment changes, so the system is always near steady state. Therefore, the competitive interactions between the strains dominate the abundance dynamics. This leads to negative correlations between members of the same guild because they consume very similar resources (Fig. 2D, right panels). This can also be seen in the eigenvalues and corresponding eigenvectors (Fig. 2E, right panels) from PCA on the dynamics in this limit, which are the inverse of the those in the fast fluctuation limit. The eigenvectors encoding the guild structure correspond to the two smallest eigenvalues (Figs 2E and G). This decrease in variance explained by the coordinated dynamics of the guilds shows that the response is not cohesive and that the growth of strains within a guild inhibits the growth of other strains within that guild.

**Analytic calculation connects covariance to guild structure:** We sought to formalize the connection between guild structure and the strain–strain correlation matrix. We show analytically that a scaled version of the overlap matrix $O=GG^{T}$ provides an approximation to the expected covariance between strains in the community (Fig. S1; Supplementary Information). This is consistent with our empirical results (Fig. 2) and reveals the mathematical basis for the observed relationship between guild structure and strain–strain abundance correlation dynamics.

### Cohesion is set by guild structure and the rate of environmental fluctuations

After studying the effect of environmental fluctuation rate, we asked what other factors modulate the cohesion of the metabolic guilds. In addition to the timescales of environmental fluctuation noted above, we hypothesized that the cohesive response requires the well-defined block structure of the trait matrix $G$ and that cohesion may depend on the size of the guild.

**Figure 3:**
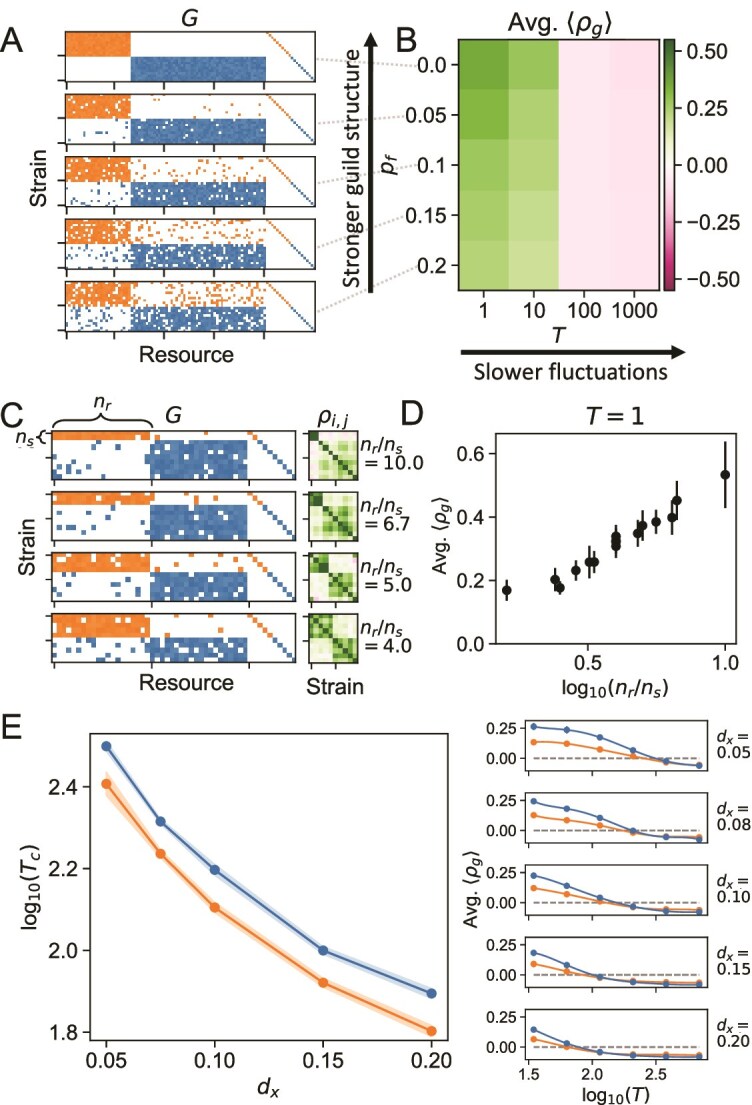
Intra-guild cohesion is driven by resource utilization structure and perturbation timescale. (A) Examples of growth rate matrices $G$ corresponding to different values of $p_{f}$ (dashed lines). (B) Average intra-guild strain–strain correlations $\langle \rho _{g} \rangle $ in simulated abundance correlation matrix (for the blue guild) averaged across 10 simulations for different values of $p_{f}$ and environmental fluctuation timescale $T$ (Methods). A higher average $\langle \rho _{g} \rangle $ indicates a higher level of cohesion within the guild. (C) Illustration of the effect of changing guild size on intra-guild cohesion. The left panels show examples of $G$ with varying sizes of the orange guild ($n_{s}$) with a fixed number of resources for that guild ($n_{r}$). The right panels show examples of corresponding strain–strain correlation matrices at $T=1$, with the same colormap as in panel B. The values of $n_{s}/n_{r}$ on the right correspond to the orange guild. (D) Increasing guild size decreases cohesion. Average $\langle \rho _{g} \rangle $ across 10 simulations as a function of the log-ratio of the number of strains to the number of resources. The error bars show the standard deviation of average intra-guild correlation $\langle \rho _{g} \rangle $. Default simulation parameters are used, and $n_{s}/n_{r}$ is varied by first fixing $n_{r}=40$ for each guild and varying $n_{s}$. Next, $n_{s} = 10$ was fixed for both guilds, and $n_{r}$ was varied. Average $\langle \rho _{g} \rangle $ is shown for both guilds. (E) Death rate sets the timescale of crossover from positive to negative intraguild correlations. The left panel shows the logarithm of the crossing timescale $\log _{10}(T_{c})$, plotted as a function of death rate $d_{x}$. The shaded region indicates uncertainty (Methods). $T_{c}$, which can be interpreted as the characteristic timescale of the system, decreases as $d_{x}$ increases. Average growth rate $\langle g \rangle $ is set to 1 by changing average uptake rate $\langle r \rangle $ to 5 and keeping yields $\gamma _{i,\alpha }$ at the default value of 0.2, so death rate can be interpreted as a fraction of the growth rate. All other parameter values are set to the default values (Methods). The right panels show the average $\langle \rho _{g} \rangle $ as a function of the logarithm of the environmental fluctuation timescale $\log _{10}(T)$ for different death rates $d_{x}$. The error bars indicate standard deviations across 10 simulations, and the blue lines indicate the guild with more resources, whereas the orange lines indicate the guild with fewer resources (as in panel A and Fig. 2).

To examine the effect of guild structure on cohesion, we first simulate the system described in Equation 2 while varying two parameters: the flip probability $p_{f}$, which is the probability that each growth rate $g_{i,\alpha }$ deviates from the block structure (quantifying the level of guild structure of the trait matrix $G$), and the fluctuation timescale $T=2 \pi / \langle \omega \rangle $ (Fig. 3). Thus increasing $p_{f}$ corresponds to a weakening of the guild structure. The cohesion of the community response is quantified by calculating the average intra-guild strain–strain correlations $\langle \rho _{g} \rangle $ averaged across 10 simulated correlation matrices and randomly generated environments (Fig. 3; Methods). On fast timescales ($T = 1, 10$), we see a monotonic decrease in the average correlation as $p_{f}$ increases (Fig. 3B). We conclude that the cohesion of guilds in response to environmental perturbations is modulated by the strength of the guild structure, with stronger guilds resulting in more cohesive responses at fast timescales.

After examining the effect of guild structure on guild cohesion, we asked how the size of the guild affects cohesion. We define guild size as the number of species in the block relative to the number of resources that they primarily consume, $n_{s}/n_{r}$ (Fig. 3C). We find a monotonic increase in cohesion ($\langle \rho _{g} \rangle $) as this ratio decreases (Fig. 3D). Thus, smaller guilds are more cohesive than larger guilds when normalized to the number of resources. This is likely due to higher per capita availability of block resources for smaller guilds, which leads to these resources having a larger effect on the abundance dynamics relative to independent resources. Here, such resources are in the form of diagonal private resources. To check this effect, we varied the level of the private resources relative to the block resources, and we find that increasing private resource levels decreases the magnitude of the correlation (Fig. S2), as expected. However, we cannot fully rule out other effects mediating intra-guild correlations as the size of the guild and the number of resources consumed varies.

### Death rate sets the timescale of the system

Here, we ask what biological parameters set the timescale of the ecological processes. In particular, we define this timescale as the environmental fluctuation period at which average $\langle \rho _{g} \rangle $ switched sign, $T = T_{c}$. We then repeat our simulations with $p_{f}=0.1$ for different values of the growth rate, death rate, and intra-guild variance in growth rate. The intra-guild variance in growth rate is defined as the width of the distribution from which nonzero growth rate values are drawn (Methods). We then fit the average intra-guild correlation as a function of the fluctuation period to a smoothing spline. This generates a mathematical function that smoothly interpolates between simulated values, allowing us to infer $T_{c}$ for each parameter set (Fig. 3E, right panels; Methods).

We find that the death rate sets $T_{c}$. Both guilds show decreasing $T_{c}$ as the death rate increases (Fig. 3E), with differences between guilds arising from the difference in guild size (as discussed in the previous section). In our simulations, $T_{c}$ does not depend on the growth rate or variance in intra-guild growth rate (Fig. S3A and B), but we cannot rule out the possibility in different parameter regimes. Finally, we examined whether the amplitude of resource fluctuations, $K_{A,\alpha }$, influenced either the average species–species correlation, $\langle \rho _{g} \rangle $, or the correlation timescale, $T_{c}$. We found no significant effect (Fig. S3C), suggesting that species–species correlations are sensitive to the timescale of fluctuations in $K_{\alpha }$ rather than the magnitude of those fluctuations. Overall, this result indicates that the transition from a cohesive, environmentally dominated regime to a noncohesive, competition-dominated regime is set by how quickly organisms die. Ecologically, this is intuitive: the death rate effectively sets the turnover rate of the community and therefore the timescale on which ecological processes occur [34].

In the next section, we turn to an experimental test of the theoretical principles we outline above. In particular, we use a serial batch culture approach with a synthetic bacterial community to measure abundance correlations across timescales.

### Experimental test of timescale dependence of guild cohesion

In this section, we test the proposal that intra-guild abundance dynamics are cohesive on short timescales and not cohesive on longer timescales. Experimentally, imparting sinusoidal resource fluctuations is a challenge. However, performing serial batch culture experiments, where communities are grown for fixed intervals before being diluted into fresh nutrients, is standard [35]. Therefore, we set out to design a serial batch culture experiment, with synthetic communities, where the $G$ matrix is inferred from experimental data and the environment can be manipulated. To do this, we first assayed the growth phenotypes of 20 strains on 10 distinct carbon sources [36] in monoculture. Second, we constructed a synthetic community from these isolates, and rather than fluctuating resources over time, we varied the resources present in the community. We repeated this experiment for many combinations of resources. Although these experiments are exploratory and do not purport to demonstrate the mechanism proposed above conclusively, we show below how the dynamics of this experiment exhibit qualitatively similar behavior to the situation analyzed above.

#### Timescale dependence of cohesion across environments

To relate our theoretical result to an experimental batch serial-dilution context, we redefine timescale of environmental fluctuations as the length of time that has passed following an environmental change. Intuitively, a discrete approximation to the continuous fluctuation case could consist of a sequential, step-wise, random change in the resource influx rates $K_\alpha $, with rate of change $\omega _\alpha $. This produces an ensemble of environments, where the time spent in each environment is set by the timescale $T$ (Equation 3). Therefore, we expect that the dynamics of correlations in abundances measured over ensembles of random, static environments should reflect temporal correlations measured at different timescales of fluctuation $T$, with correlation coefficients decreasing as a function of time elapsed in the set of environments just as temporal correlations decrease as a function of $T$.

To test the intuition that the time elapsed following an environmental perturbation is analogous to fluctuation timescale, we initialize our two-guild communities at equal abundances, subject them to many randomly generated environments, and calculate correlations between strain abundances across environments as a function of time. To mimic experimental serial-dilution conditions, we supply nutrients to the community in periodic batches and dilute after a fixed time. The model (Equation 2) then becomes


(4) \begin{align*}& \begin{aligned} \frac{dx_{i}}{dt} &= x_{i} \displaystyle\sum_{\alpha = 1}^{M} r_{i,\alpha} \gamma_{i,\alpha} \frac{R_\alpha}{R_\alpha + R_{0}}\\ \frac{dR_\alpha}{dt} &= - \displaystyle\sum_{i = 1}^{N} r_{i,\alpha} \frac{R_\alpha}{R_\alpha + R_{0}}x_{i} \\ \end{aligned}\end{align*}

with initial conditions defined for each batch cycle by


(5) \begin{align*}& \begin{aligned} x_{i}^{c}(0) &= x_{i}^{(c-1)}(t_{D})/D \\ R_{\alpha}^{c}(0) &= K_{\alpha}^{b} (D-1) / D + R_{\alpha}^{(c-1)}(t_{D}) / D, \\ \end{aligned}\end{align*}

where $c$ indexes the cycle, $D$ is the dilution factor during serial dilution (taking the place of death rates $d_{x}$ and $d_{R}$ in Equation 2), mimicking a dilution constant in batch culture experiments [1]. $K_{\alpha }^{b}$ is the concentration of resource $\alpha $ supplied in the fresh media at the beginning of each cycle. $x_{i}^{c}(0)$ and $x_{i}^{c}(t_{D})$ are the values at the beginning and end of cycle $c$. $R_\alpha ^{c}$(t) is defined in the same way.

We numerically integrate this model for randomly generated versions of the two guild communities used above (Fig. S4A and B; Methods). Next, we calculate strain–strain abundance correlations across an ensemble of 100 randomly generated environments as a function of serial dilution cycle (Methods). Just as average intra-guild correlations decrease as a function of time in the continuous case (Figs 2 and 3), correlations across environments in the batch case decrease with number of dilution cycles (Fig. S4C). This suggests that responses to differing fluctuation rates and time spent in a new environment are analogous, and that we can generalize our theoretical results to an experimentally tractable batch case.

#### Correlation dynamics *in vitro* are consistent with theoretical and *in silico* expectations

To test the implications of our theoretical results, we conducted an experiment to measure correlations between strains competing for resources across time. We grew a synthetic community consisting of 20 strains with known carbon utilization capabilities [36] for nine cycles in serial batch culture in 32 different, randomly generated environments. Next, we used 16S rRNA gene sequencing with a spike-in to measure the absolute abundances of each strain at the end of each cycle (Methods).

**Figure 4:**
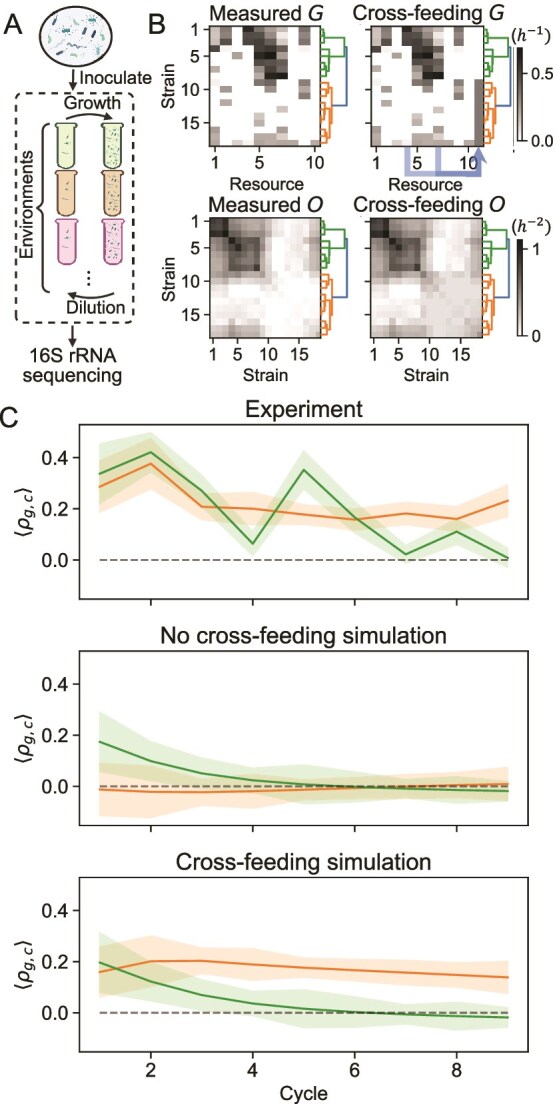
Experimental demonstration of decreasing guild cohesion over time during serial batch culture. (A) Experimental schematic. A synthetic community with 20 strains was inoculated into 32 environments, each comprised of a number of randomly chosen carbon resources. This community was passaged for nine batch growth cycles, with a 10-fold dilution into fresh media at the end of each cycle. (B) Prior to the experiment, the $G$ matrix was estimated by growing each strain independently on each of the 10 carbon sources (Methods). In the top left panel, entries are growth rates measured via OD in time in a plate reader, with the colormap varying linearly from $0$ to $\text{max}(G) = 0.7 \text{ h}^{-1}$. Hierarchical clustering on the 18 strains with measured abundances in all cycles is used to identify guilds (green and orange branches in dendrogram) on this matrix. The top right panel shows the $G$ matrix used to test the cross-feeding hypothesis using the same colormap. In particular, a hypothetical secondary metabolite consumed by the orange guild is added in an additional 11th column. The blue arrows indicate the provided resources that are converted to this secondary metabolite in this scenario. The bottom row shows inferred overlap matrices $O$ of the synthetic communities. Guilds are shown by the dendrograms, and the colormaps are linear from $0$ to $\text{max}(O) = 1.1 \text{ h}^{-2}$. (C) Timeseries of the experimental (top plot, measured by sequencing; Methods) and simulated (middle and bottom plots) average intra-guild correlation for the synthetic community in the experimental environments. Variance is calculated by resampling with replacement and shown by the shaded region (Methods). The green and orange colors correspond to the green and orange guilds, respectively (panel B). In the simulation, parameters $D$ and $K_{\alpha }^{b}$ are matched to experimental values (Methods). The panel in the middle shows results from a simulation with no cross-feeding interactions, whereas the bottom plot results from a simulation with hypothesized cross-feeding interactions.

To compare the experiment with the *in silico* expectation, we first need to define experimental guilds. For the 18 strains with measured abundances in all cycles, we use hierarchical clustering to identify two metabolic guilds (Fig. 4B; Methods). Next, we integrate Equation 4 with the resources provided in the experiment and the inferred $G$ matrix. Finally, we plot the average intra-guild correlation $\langle \rho _{g,c} \rangle $ for each inferred guild $g$ at each cycle $c$ in both the simulation and experiment.


(6) \begin{align*}& \langle \rho_{g,c} \rangle = \frac{\sum_{i \in g} \sum_{j \in g, j <i} \rho_{i,j,c}}{N_{g}}\end{align*}

where $\rho _{i,j,c}$ is the Pearson’s correlation coefficient across environments between strains $i$ and $j$ at cycle $c$ (Methods), and $N_{g}$ is the number of strain pairs in the guild.

For the guild that consists of strains that can utilize many of the carbon resources (Fig. 4C top plot, green curves), we observe the characteristic decrease in $\langle \rho _{g,c} \rangle $ associated with metabolic guilds interacting via resource competition. This is consistent with our theoretical expectation (Fig. S4C). This recapitulates the basic intuition that strains within a guild are positively correlated for short timescales of environmental variation and the converse (Fig. 2).

For the other guild (Fig. 4C top plot, orange curves), we do not see a significant decrease in $\langle \rho _{g,c} \rangle $ across cycles in either the simulation or experiment. Examination of the resource utilization of the strains in this guild, however, shows that they do not grow quickly on *any* resources relative to the strains in the green guild, and that the resources they do consume are also consumed by this guild (Fig. 4B). This suggests that these strains are unlikely to be able to survive by competing for resources. Although this is the case *in silico* (Fig. S5A), strains in the orange guild are able to survive *in vitro* (Fig. S5B). To account for this discrepancy between experiment and simulation, we recall that the simulation assumes that resource competition is the only mechanism of interaction between strains in the community. We speculate, therefore, that strains in the orange guild survive due to cross-feeding interactions with strains in the green guild, perhaps mediated by overflow metabolism [14]. A fine-grained examination of correlations between strains provides support for this idea. On long timescales, strains in opposite guilds (Fig. S6A diagonal, bold blue lines in Fig. S6B) and strains that are both within the orange guild (Fig. S6A diagonal, bold orange lines in Fig. S6B) are often highly correlated, but strains that are both within the green guild are not often correlated (Fig. S6A diagonal, green lines in Fig. S6B). This is consistent with competition dominating intra-green guild correlations on long timescales, but orange guild members relying on resources produced by green guild members, resulting in correlations with these green guild members and other orange guild members. In the next section, we add a cross-feeding interaction to the simulation of the synthetic community to test this hypothesis more carefully.

#### Cross-feeding interactions *in silico* reproduce qualitative features of *in vitro* intra-guild correlation dynamics

To test the hypothesis that cross-feeding interactions are sufficient to reproduce the qualitative features of the experimental intra-guild correlation dynamics, we add cross-feeding interactions to our models of community abundance dynamics. First, we use a well-established cross-feeding formalism [34] to show that cross-feeding leads to inter-guild correlations on long timescales in the theoretical continuous growth scenario considered in the first half the manuscript (Fig. S7; Supplementary Information). Next, we add the cross-feeding interactions explicitly to the simulation of the synthetic community experiment (Fig. 4). We hypothesize the existence of an additional resource that is never supplied directly but is produced when resources 4 and 7 are consumed (Fig. 4B, “cross-feeding $G$;” Methods). Together, these metabolic transfers constitute *inter-*guild cross-feeding (resource 4 is consumed almost exclusively by the green guild, and its secondary metabolite is taken up by the orange guild) and a combination of *inter-* and *intra-*guild cross-feeding (resource 7 is consumed by both the green and orange guilds, and its secondary metabolite is taken up by the orange guild). Modification of Equation 4 to add these interactions yields


(7) \begin{align*}& \begin{aligned} \frac{dx_{i}}{dt} &= x_{i} \displaystyle\sum_{\alpha = 1}^{M} r_{i,\alpha} \gamma_{i,\alpha} (1 - l_{\alpha})\frac{R_\alpha}{R_\alpha + R_{0}}\\ \frac{dR_\alpha}{dt} &= \displaystyle\sum_{i = 1}^{N} \Bigg[ -r_{i,\alpha} \frac{R_\alpha}{R_\alpha + R_{0}}x_{i} + \displaystyle\sum_{\beta = 1}^{M} r_{i,\beta} \frac{R_{\beta}}{R_{\beta} + R_{0}}x_{i} l_{\beta} T_{\alpha,\beta} \Bigg] \\ \end{aligned}\end{align*}

where the leakage vector $\vec{l}$ ($l_{4}=l_{7}=0.25$, all other $l_{\alpha }=0$) and the transformation matrix $T$ ($T_{11,4}=T_{11,7}=1$, all other $T_{\alpha ,\beta } = 0$) encode the cross-feeding interactions (Methods; Supplementary Information). All other parameter values are kept the same as in the non-cross-feeding case. We emphasize that the choice of these values is speculative and is not based on any experimental characterization of metabolic byproducts of the strains involved.

In this hypothetical cross-feeding scenario, we are able to qualitatively reproduce the long-term positive intra-guild correlation in the orange guild, while maintaining the decrease in intra-guild correlation in the green guild over time (Fig. 4C, top and bottom plots). We expect that inter-guild cross-feeding is necessary to allow the orange guild strains to survive and speculate that intra-guild cross-feeding may promote long-term intra-guild correlations. Overall, this result demonstrates that cross-feeding interactions are sufficient to reproduce the qualitative features of the experimental result that are inconsistent with a strictly resource competition-based model. However, we emphasize that this is *only* a sufficiency statement, as we do not have adequate data to constrain and quantitatively evaluate a cross-feeding model.

## Discussion

Here, we have characterized the response of microbial communities composed of metabolic guilds to environmental fluctuations. We demonstrate that fast environmental fluctuations excite a cohesive response within metabolic guilds and that cohesion is lost for slow environmental fluctuations.

The changing cohesion of metabolic guilds with timescales of fluctuations has important implications for understanding community dynamics in the wild. For example, it is routine to study correlations in abundances of microbes across time and space [37]. Interpreting correlated abundance dynamics is a long-term challenge in microbial ecology [38, 39]. We show that the sign of correlations in abundance dynamics can vary strongly with the timescale of environmental variation driving abundance dynamics. This is true when timescale is defined as either: (1) the rate of environmental fluctuation (Figs 2 and 3) or (2) time after environmental perturbation (Figs S4 and 4). This means that care must be used in interpreting when abundance correlations might reflect ecological associations [40]. The observation also offers an opportunity to understand metabolic guild structure in communities and to expose underlying structural properties that drive community function.

### Relevance to macroecological patterns

Several recent studies have revealed conserved statistical properties in bacterial abundance distributions across diverse microbiomes [41–43]. These studies show that simple models of population dynamics driven by random environmental fluctuations can recapitulate patterns in average relative abundances and variation in abundances across replicate communities. Here, we discuss the relevance of these observations to the present findings regarding guild cohesion.

One study describes gut microbiome data using a consumer-resource model that coarse-grains all members of a guild into a single population growing a common set of resources [43]. In the model, resource preferences at the guild level are randomly sampled, and resources fluctuate. This model captures statistics of abundances when sequencing data are coarse-grained at the family level, which roughly corresponds to metabolic guilds. In this picture, intra-guild dynamics are not considered. Pairwise abundance correlations between guilds in the data were observed to be centered around zero with a broad range. In view of our findings, this might arise from fast resource fluctuations and significant overlap between guilds in resource utilization or cross-feeding between guilds.

More recent work extends this result to observed phylogenetic correlations for microbiomes across a range of habitats [42]. It is observed that more closely related taxa exhibit correlated fluctuations. The analysis of empirical fluctuations is performed at the operational taxonomic unit (OTU) level, a finer taxonomic level than that reported previously [43]. At the OTU level, we expect resource overlap between closely related taxa. We observe no statistically significant phylogenetic correlation in our synthetic community data (Fig. S8), but this may be due to limited phylogenetic diversity in our consortium. Nonetheless, the more recent work ([42]) uses a model to test whether the observed patterns arise from resource competition or from environmental factors that affect growth independently of resources. This shows that the patterns are reproduced when closely related taxa experience correlated growth rate perturbations arising from resources and abiotic environmental fluctuations (e.g. temperature, pH). Therefore, one explanation for this observation in light of our results is that these correlations arise from a combination of correlated responses to resources in the fast resource fluctuation regime and correlated responses to abiotic perturbations. It remains unclear how this mechanism can hold across microbiomes in vastly different niches, such as the gut or soil, where growth rates and resource availabilities differ by orders of magnitude.

### Implications for experimental determination of metabolic guilds

In addition to providing insight into natural ecological processes, this work informs experimental design. In particular, the inference of metabolic guilds has recently been a topic of interest in microbial ecology and microbiology [44–46]. Identification of metabolic guilds in microbial communities is valuable because it can enable coarse-graining communities using effective groups of taxa [2, 47, 48]. This coarse-graining provides a low-dimensional description of communities that can enable predictions of community dynamics and function.

A common approach to inferring guilds is to perform serial dilution enrichments to identify strains that perform well in an environmental condition of interest [35]. Our theoretical results suggest that the observed correlations between strains in such an experiment depend on the timescale over which those correlations are observed. Thus, ideally, one would observe correlated responses of groups over short timescales. We proposed and tested an experimental procedure for identifying metabolic guilds via serial-dilution experiments. One can imagine this approach being scaled up by performing short-term enrichment experiments on complex communities in diverse environments [49]. In these contexts, our approach might help define metabolically cohesive units within the community.

### Extension to cross-feeding interactions and alternative guild definitions

Here, we largely restricted ourselves to consideration of resource competition. Recent work suggests that such interactions dominate in many contexts [43, 50]. However, it is the case that metabolic byproducts from one organism can be used by another, and these cross-feeding interactions are qualitatively distinct from competition [1, 18]. Intriguingly, we find that simulations with cross-feeding interactions give rise to positive inter- and intra-guild correlations for *all* fluctuation timescales (Fig. 4C; Supplementary Information). This is in contrast to the case with only competition, where we observed positive intra-guild correlations on short timescales, negative intra-guild correlations on long timescales, and uncorrelated inter-guild dynamics across timescales. Furthermore, in our serial batch culture experiment, correlations lagged by three cycles seem to be stronger than other time-lagged correlations, perhaps suggesting a slow build-up of metabolites that are consumed after reaching some critical value (Fig. S6A, off-diagonal correlation matrices). Together, these observations raise the possibility that different classes of interactions can be identified by studying correlation functions (e.g. [40]) across timescales of environmental fluctuation. The role of cross-feeding in driving dynamics between guilds is an important avenue for future work.

Another potential extension to the work presented here is the consideration of different guild definitions. Although our working definition of guilds based on resource preference is common in microbial ecology [15, 19, 29], alternative definitions are possible. For instance, perhaps a phenomenological, dynamic definition of a guild as a species with correlated abundances would be preferable.

As an alternative to guild definitions based solely on resource preference, classical ecological literature emphasizes a similar resource exploitation strategy as an additional consideration for guild classification [51]. For instance, strains with similar growth rates on a class of resources may nevertheless have varying uptake rates ($r$), yields ($\gamma $), and affinities ($R_{0}$), perhaps reflecting ecological strategies constrained by physiological tradeoffs [52–55]. Unlike the cross-feeding case, the qualitative patterns we observe with simple resource preferences should also apply to guilds defined by resource strategies. For instance, if the environment becomes more favorable for a given strategy, all strains using that strategy should initially benefit, producing a short-term, correlated intra-guild response. On longer timescales, however, resource competition should still dominate the dynamics, leading to an anticorrelated intra-guild response. Although we expect the qualitative pattern to hold, a quantitative analysis of how guild cohesion depends on timescale, across different guild definitions, would be an interesting extension of this work.

### Impact of evolution on guild cohesion

It is important to consider evolution when evaluating guild cohesion. In the current work, we have assumed that microbial metabolic phenotypes are fixed, even over thousands of generations. Although evolution has been shown to break this assumption in laboratory conditions on these timescales [56, 57], species in natural communities exhibit evidence of long-term evolutionary stability [58]. In any case, we again expect the qualitative pattern to hold, even in the presence of evolution: fast timescales should be unaffected, and strains with broadly similar resource preferences are still likely to be anticorrelated due to resource competition. If a strain acquires a mutation to become more competitive in the slowly fluctuating case, its increase in abundance would come at the expense of other members of its guild, and the intra-guild response would remain anticorrelated. Quantitative examination of the effect of evolution on guild-level responses to fluctuating environments is another worthwhile extension of the current work.

## Supplementary Material

Figure_S1_wraf186

Figure_S2_wraf186

Figure_S3_wraf186

Figure_S4_wraf186

Figure_S5_wraf186

Figure_S6_wraf186

Figure_S7_wraf186

Figure_S8_wraf186

SI_updated_fig_captions_wraf186

SI_updated_refs_wraf186

## Data Availability

Code and data associated with this manuscript, including code and data sufficient to reproduce all figures, are publicly available at 10.17605/OSF.IO/J8S2V. Raw sequence reads for batch culture experiments are deposited under National Center for Biotechnology Information BioProject ID PRJNA1240007.
