# Supplementary material for: Timescale of environmental change modulates metabolic guild cohesion in microbial communities
Source: ISME J. 2025 Aug 22;19(1):wraf186. doi: 10.1093/ismejo/wraf186 (PMC12448442; doi:10.1093/ismejo/wraf186)
Supplement: Figure_S1_wraf186 [file figure_s1_wraf186.pdf]

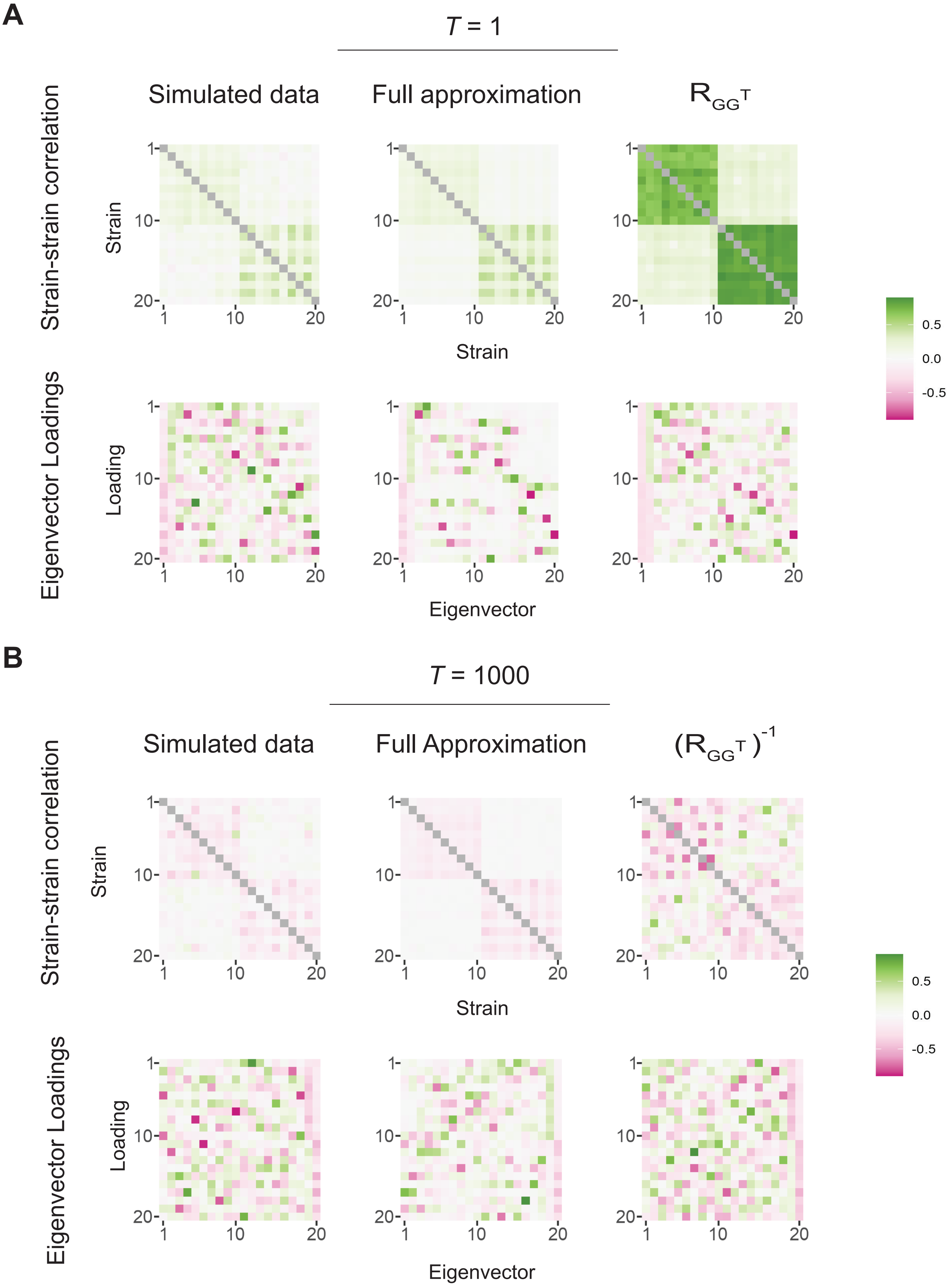

A
T = 1
Simulated data
Full approximation
R_GGT
Strain-strain correlation
Strain
Eigenvector Loadings
Loading
Eigenvector
0.5
0.0
-0.5
B
T = 1000
Simulated data
Full Approximation
(R_GGT)^-1
Strain-strain correlation
Strain
Eigenvector Loadings
Loading
Eigenvector
0.5
0.0
-0.5
