# Supplementary figures and images for: Timescale of environmental change modulates metabolic guild cohesion in microbial communities
Source: ISME J. 2025 Aug 22;19(1):wraf186. doi: 10.1093/ismejo/wraf186 (PMC12448442; doi:10.1093/ismejo/wraf186)

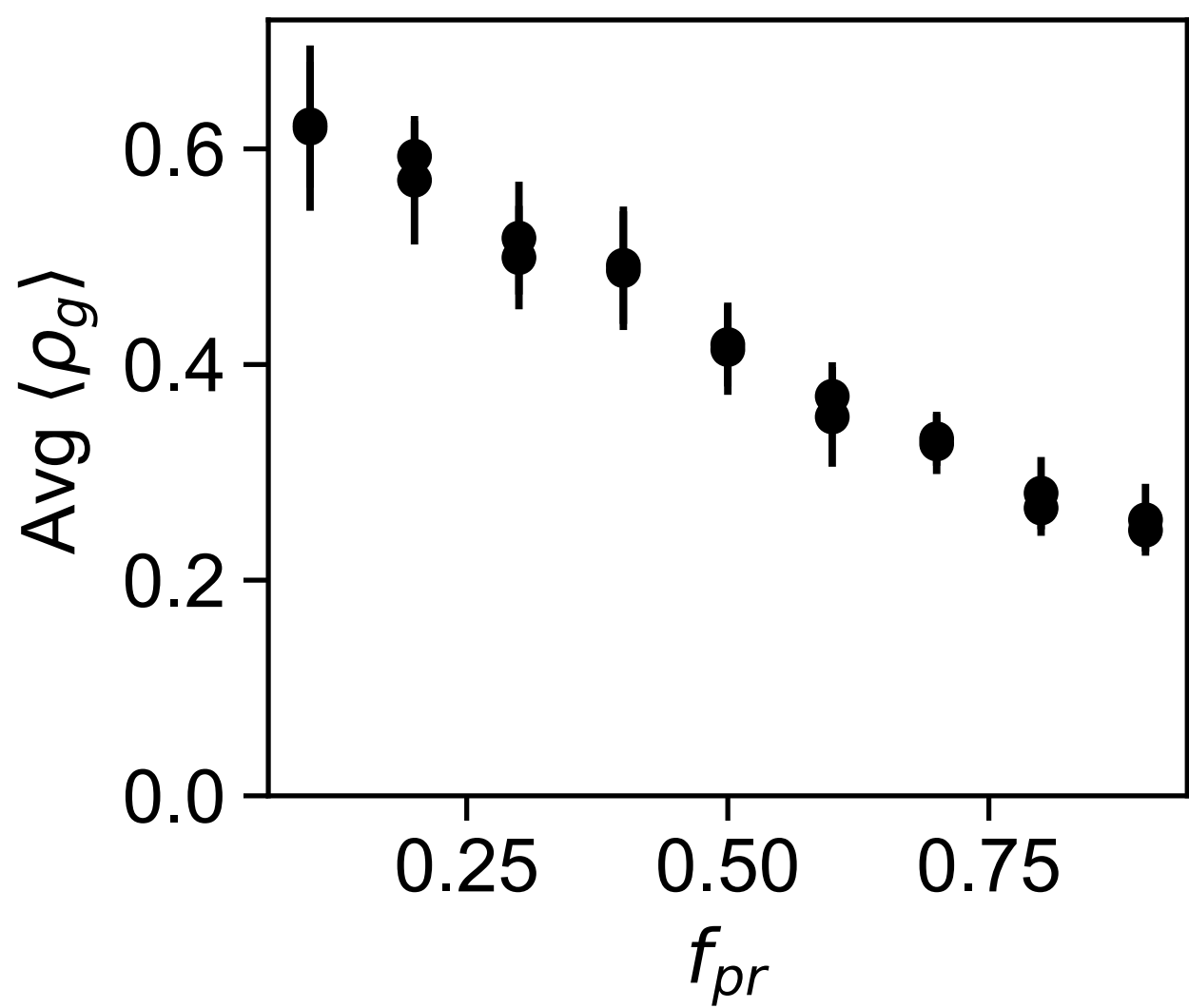

Supplement: Figure_S2_wraf186 [file figure_s2_wraf186.pdf]

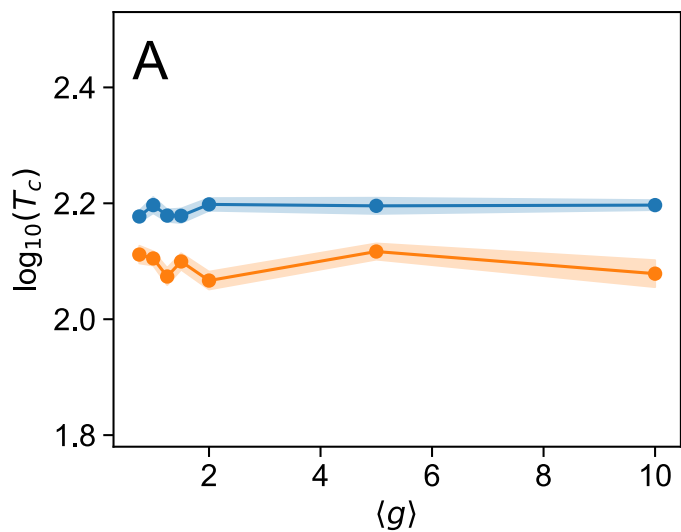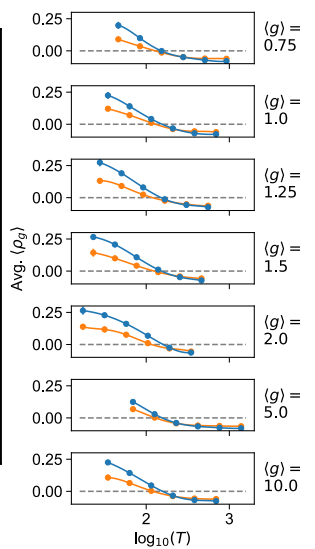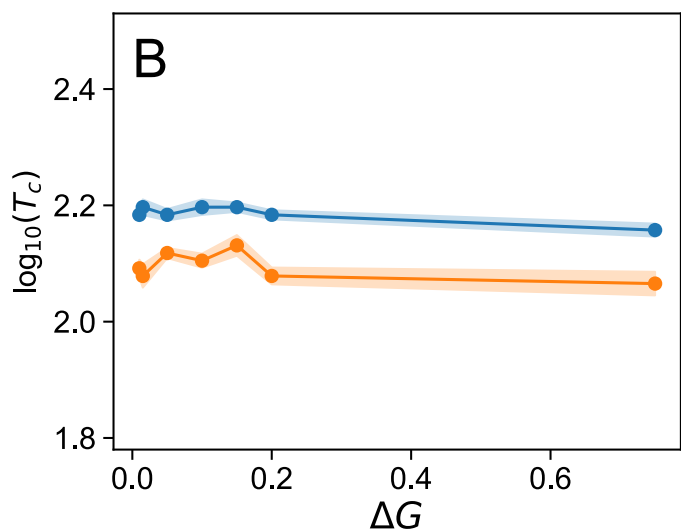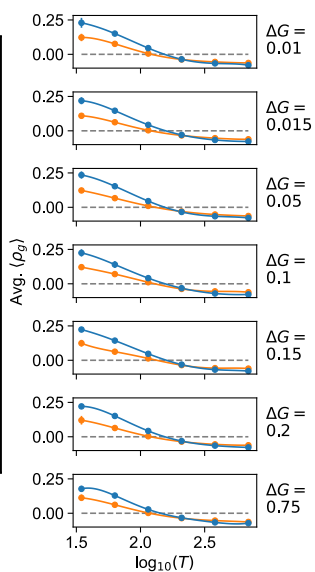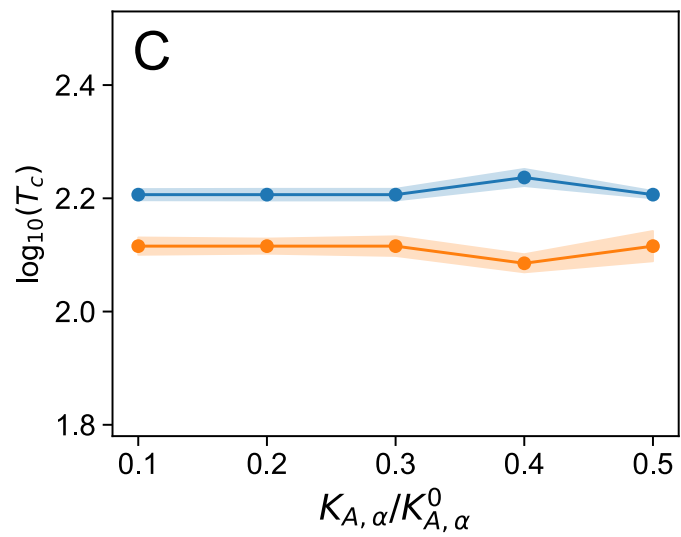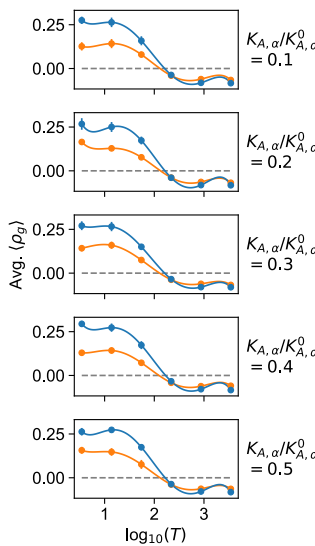

Supplement: Figure_S3_wraf186 [file figure_s3_wraf186.pdf]

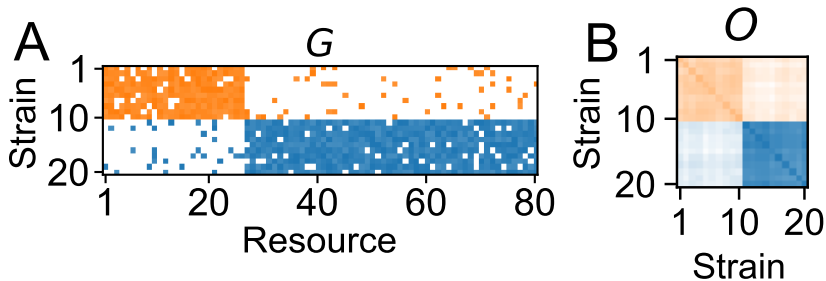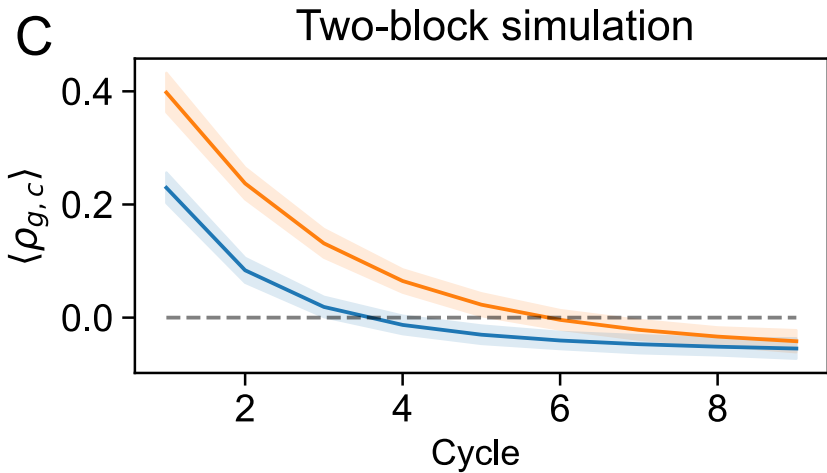

Supplement: Figure_S4_wraf186 [file figure_s4_wraf186.pdf]

## Experiment

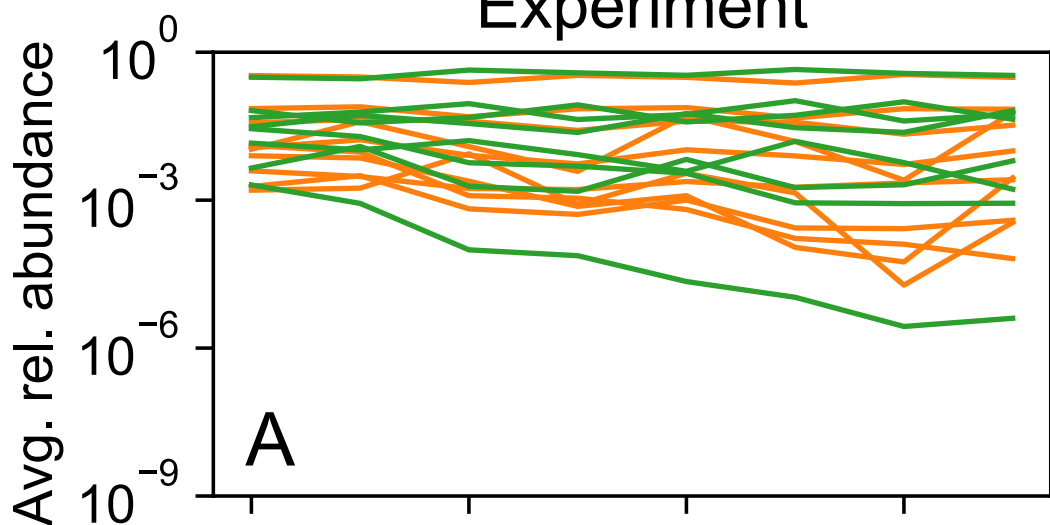

## No cross-feeding simulation

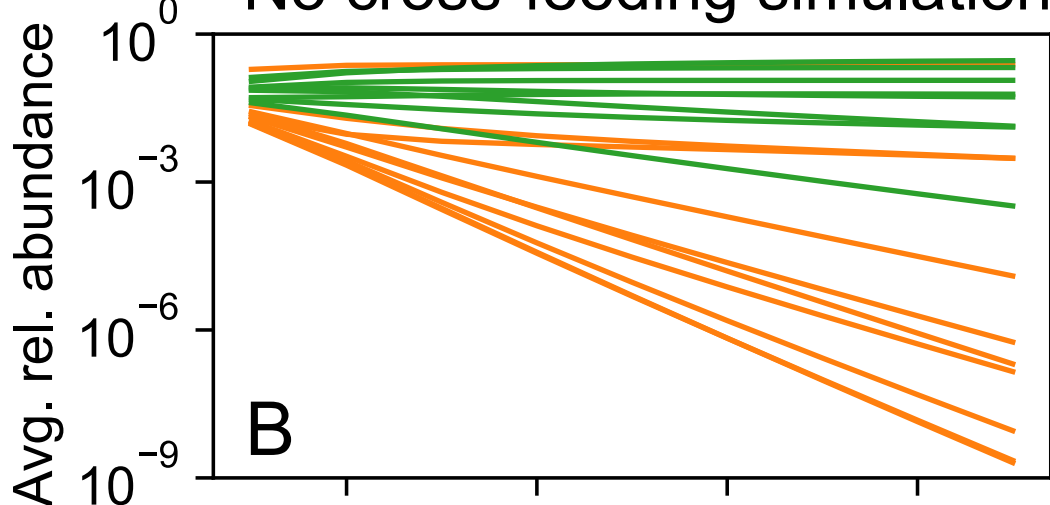

## Cross-feeding simulation

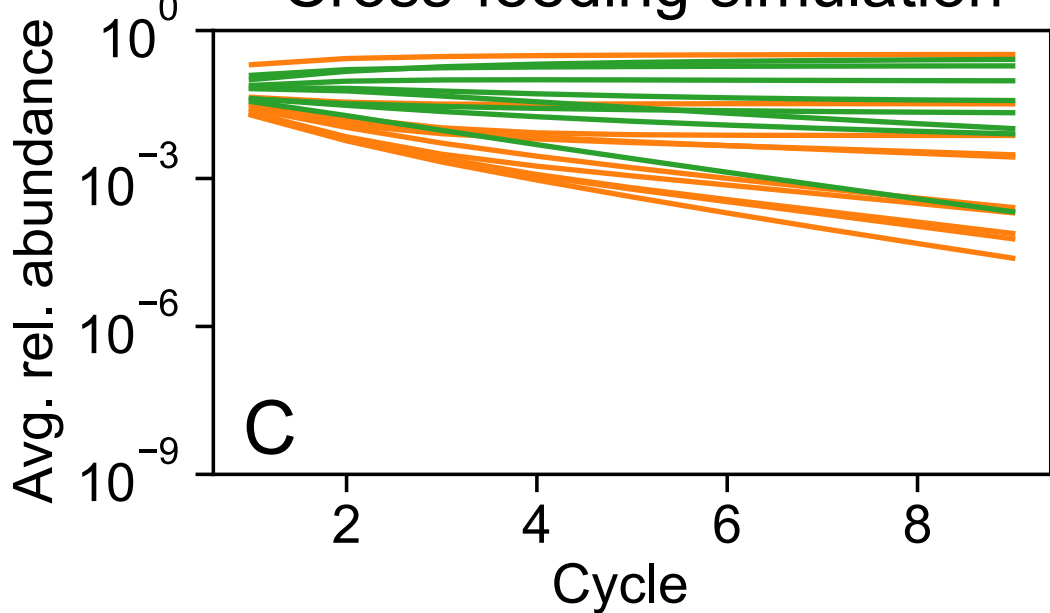

Supplement: Figure_S5_wraf186 [file figure_s5_wraf186.pdf]

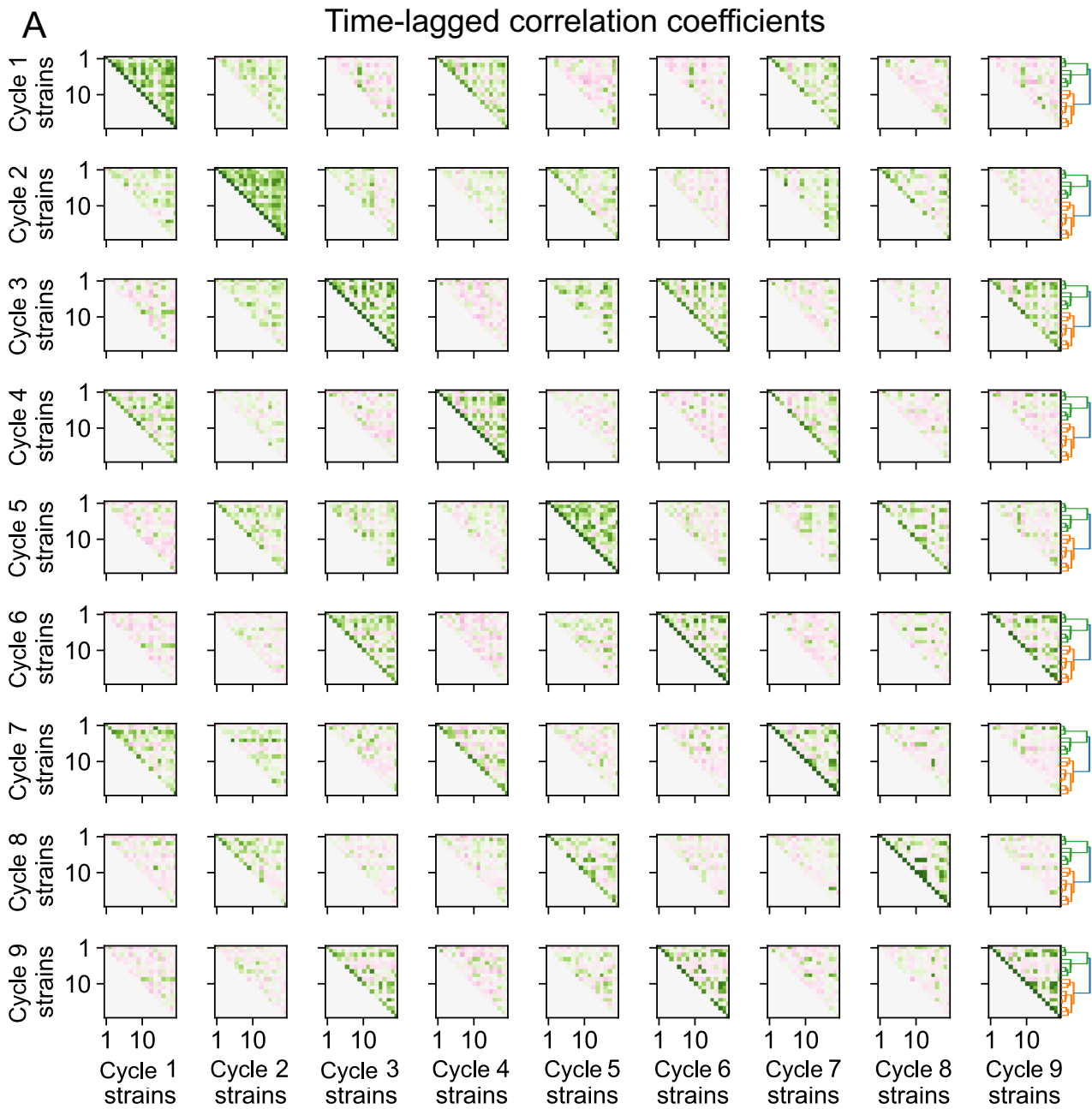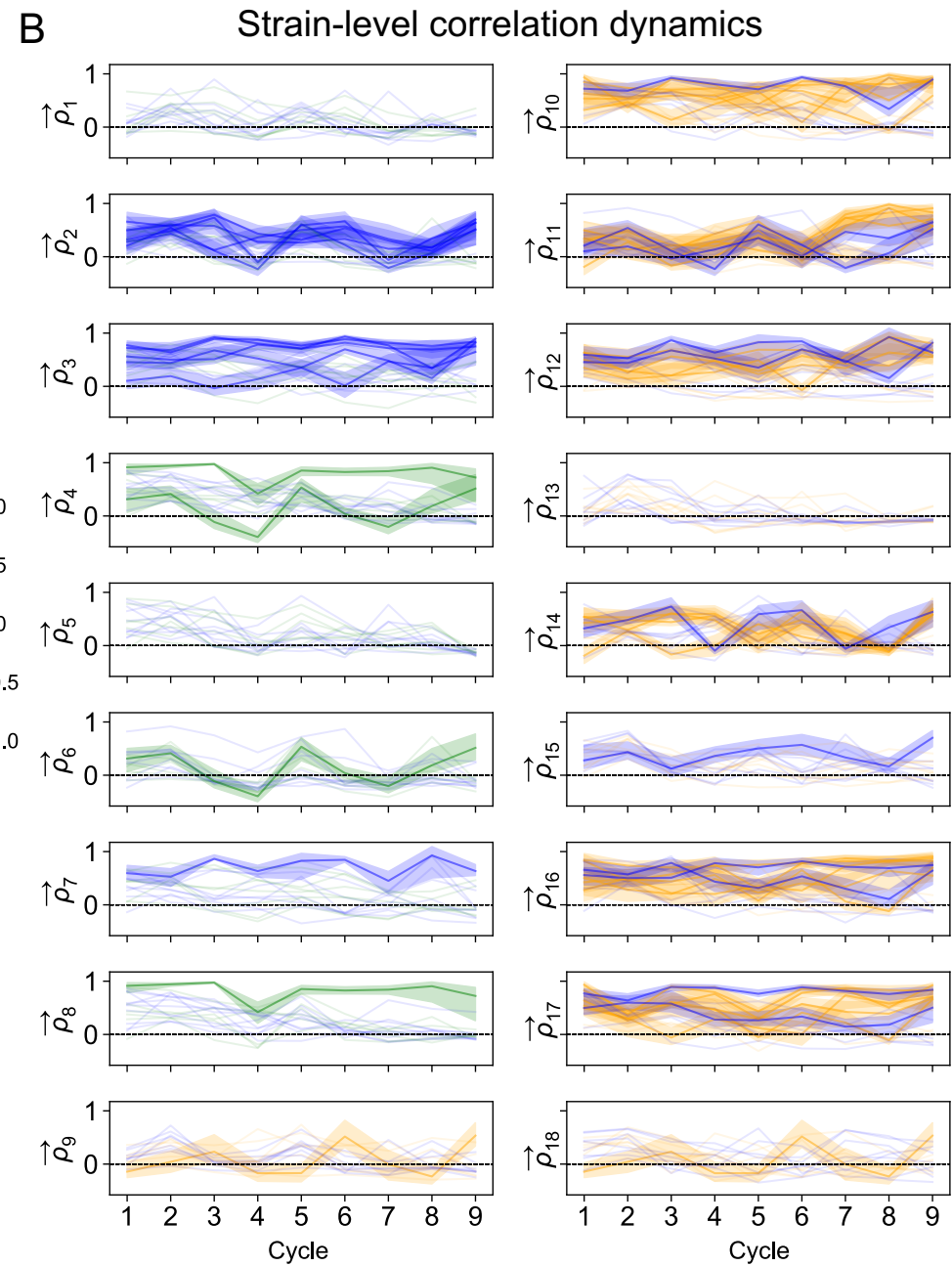

Supplement: Figure_S6_wraf186 [file figure_s6_wraf186.pdf]

$$\rho_{phylo} = -0.09$$

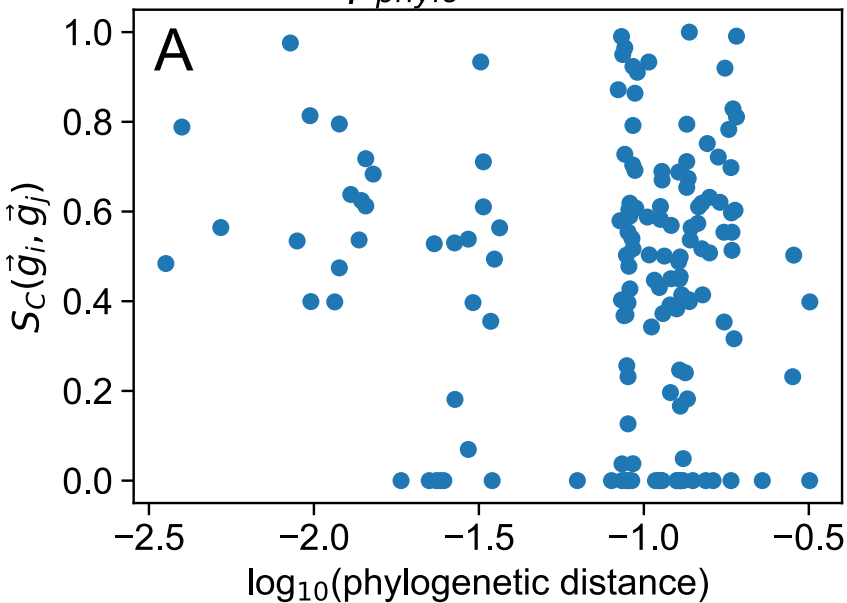

95% CI: [-0.23, 0.05],  $P = 0.1$

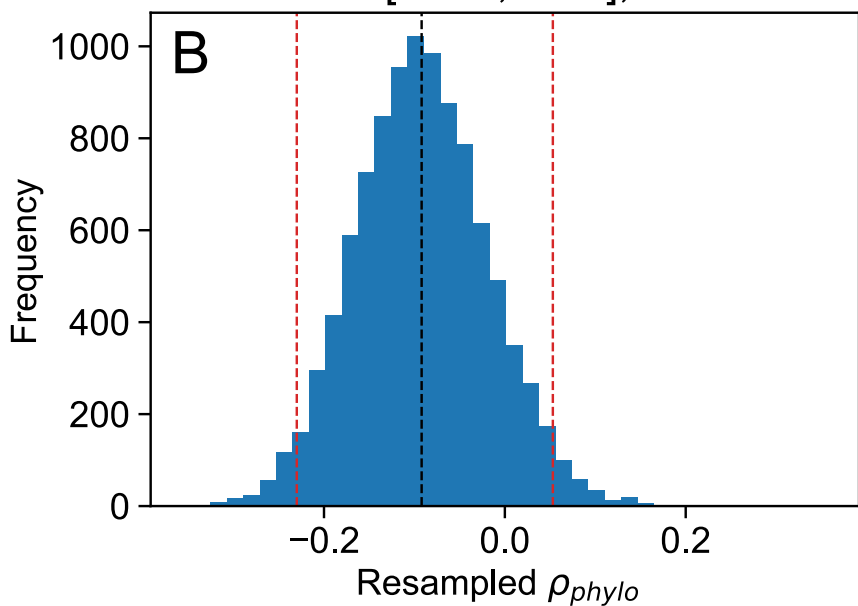

Supplement: Figure_S8_wraf186 [file figure_s8_wraf186.pdf]
