# Supplementary material for: Timescale of environmental change modulates metabolic guild cohesion in microbial communities
Source: ISME J. 2025 Aug 22;19(1):wraf186. doi: 10.1093/ismejo/wraf186 (PMC12448442; doi:10.1093/ismejo/wraf186)
Supplement: SI_updated_fig_captions_wraf186 [file si_updated_fig_captions_wraf186.pdf]

Supplementary Figure Captions

Figure S1: **Analytic derivations provide effective approximations for strain-strain correlation matrices across timescales of environmental fluctuation.** **(A)** For fast environmental fluctuations ($T = 1$ doubling time), strain-strain correlation matrices and eigenvector loadings are shown for simulated data (same as in Fig. 2 for $T = 1$), the full analytic approximation derived from Eq. 29, and the simplified approximation R_{GG^T}. The full analytic approximation is given by the correlation matrix corresponding to the inverse of the covariance matrix described in Eq. 29, whereas the simplified approximation R_{GG^T} is the correlation matrix corresponding to the overlap matrix GG^T. The strain-strain correlations show that the full analytic approximation provides excellent agreement with the data, whereas the simplified approximation still reflects the correlated intraguild response despite greater entry-wise deviations. In all cases, eigenvectors corresponding to the two largest eigenvalues reflect the coherent response within guilds. Diagonal elements of species-species correlation matrices are masked in gray to better visualize the smaller-magnitude off-diagonal correlations. **(B)** Same as A, for slow environmental fluctuations ($T = 1000$ doubling times), and with the full analytic approximation given by the correlation matrix corresponding to the covariance matrix described in Eq. 29, and the simplified approximation $R^{-1}_{GG^T}$, the correlation matrix corresponding to $(GG^T)^{-1}$, the inverse of the overlap matrix. Although the full approximation provides better agreement to simulated strain-strain correlations, the simplified $R^{-1}_{GG^T}$ approximation nevertheless captures the predominant correlation structure of anti-correlated responses within guilds. In all cases, eigenvectors corresponding to the two smallest eigenvalues reflect the guild structure. (Simulated data are again the same as in Fig. 2 for $T = 1000$.)

Figure S2: **Private resource levels modulate magnitude of species-species correlations.** Private resource levels are varied via the parameter f_{pr}, where the $K_{\alpha \in \text{private resources}} = f_{pr} K_{\alpha \in \text{block resources}}$. Error bars show standard deviation across $n = 10$ simulations performed using default parameters and two guilds of 10 species and 40 resources.

Figure S3: **Growth rate and resource amplitude do not set timescale. (A)** Average growth rate $\langle g \rangle$ does not set T_c . The left panel shows the logarithm of the crossing timescale T_c, plotted as a function of $\langle g \rangle$. $\langle g \rangle$ is varied by varying $\langle r \rangle$, setting $\Delta G = 0.1$ and all other parameter values to the default values (Methods). The shaded region shows uncertainty, calculated via resampling simulations with replacement (10 simulations, Methods). The right panels show the intra-guild correlation $\langle \rho_g \rangle$, averaged across simulations, as a function of the log of the environmental fluctuation timescale T for different values of $\langle g \rangle$. Error bars indicate standard deviation over simulations. **(B)** Variance in the growth rate matrix ΔG does not set T_c. The left panel shows $\log(T_c)$, plotted as a function of ΔG. Average growth rate $\langle g \rangle$ is set to 1 by changing average uptake rate $\langle r \rangle$ to 5 and keeping yields $\gamma_{i,\alpha}$ at the default value of 0.2, so ΔG can be interpreted as a fraction of growth rate (Methods). The shaded region shows uncertainty, calculated via resampling simulations with replacement (10 simulations, Methods). The right panels show the average $\langle \rho_g \rangle$ as a function of T for different values of ΔG. Error bars indicate standard deviation over simulations, and variance among intra-guild strain-strain correlation coefficients does not depend on ΔG. **(C)** Resource fluctuation amplitude $K_{A,\alpha}$ does not set T_c. The left panel shows $\log(T_c)$, plotted as a function of $K_{A,\alpha}$, normalized by the default $K^0_{A,\alpha} = 10$. The shaded region shows uncertainty, calculated via resampling simulations with replacement (6 simulations, Methods). The right panels show the average $\langle \rho_g \rangle$ as a function of T for different values of $K_{A,\alpha}$. Error bars indicate standard deviation over simulations.

Figure S4: **Dynamic dependence of guild cohesion in batch growth conditions.** Experimental batch culture growth is simulated in a two-block community by numerical integration of Eq. 4 with default batch simulation parameters (Methods). Parameters differ from the simulation of the experiment (Fig. 4E), but reproduce qualitatively the behavior seen in the experimental simulation and experiment. **(A,B)** G and O matrices. Private resources are omitted from G in the batch simulations. **(C)** Timeseries of the simulated average intra-guild correlation at each cycle across environments. To generate environments, each initial resource value $K_{B,\alpha}$ was set to either 0 or the default value of 1, each with probability 0.5 (Methods). Variance is calculated by resampling with replacement (Methods) and shown by the shaded region. Orange and blue correspond to the orange and blue guilds, respectively (panels A and B).

Figure S5: **Average strain relative abundance by guild. (A,B,C)** Average relative abundance across environments for each synthetic community strain in the green and orange guilds in the experiment (corresponding to the topmost plot in panel C in Fig. 4), simulation without cross-feeding (panel B, corresponding to the middle plot in panel C in Fig. 4), and simulation with cross-feeding (panel B, corresponding to the bottom plot in panel C in Fig. 4). The simulations use the experimental growth rate matrix G, along with the experimental environments (Methods).

Figure S6: **Emergence of inter-guild correlation on long timescales (A)** Upper triangular strain-strain correlation matrices $\rho_{i,j,c}$ during the experiment. Correlations are calculated based on absolute abundances inferred at the end of each cycle from sequencing with an experimental spike-in (Methods). Diagonal matrices show same-cycle correlations used to calculate average correlations (Fig. 4C), and off-diagonal matrices show across-cycle correlations. The dendrogram indicates the guilds inferred from experimental growth rate data via hierarchical clustering. **(B)** For each strain (subscript), correlation coefficients with all other strains are plotted across cycles. Intra-guild correlations are colored according to the color of the guild, inter-guild correlations are colored blue, and pairs of strains with correlation coefficient > 0.5 at cycle 9 are bolded. Envelopes show one standard deviation across an ensemble of correlation coefficients calculated from experimental environments resampled with replacement.

Figure S7: **Cross-feeding gives rise to persistent positive correlations between cross-feeding guilds. (A,D)** G matrix for the trophic (A) and specific (D) cross-feeding simulations (Eq. 31; generated and displayed as in Fig. 2A). **(B,E)** Cross-feeding matrix T for the trophic (B) and specific (E) cross-feeding simulations. For (B), resources 1-40 are transformed into resources 41-80 and excreted. For (E), resources 41-50 are transformed into resources 51-60 and excreted. In both, the colormap varies linearly from 0 to 1. **(C,F)** Pearson's correlation coefficient for the abundance dynamics of each strain pair is shown at each fluctuation timescale (as in Fig. 2D) for the trophic (C) and specific (F) cross-feeding simulations. In the trophic case (C), inter-guild positive correlations persist even for slow environmental fluctuations, whereas the sign of intra-guild correlations goes from positive to negative correlations as environmental fluctuations slow. In the specific case (F), strong inter-guild positive correlations persist even for slow environmental fluctuations for cross-feeding partners, whereas the sign of intra-guild correlations goes from positive to negative correlations as environmental fluctuations slow.

Figure S8: **Synthetic community traits are not correlated with phylogeny. (A)** Cosine similarity S_C in growth rate vectors $\vec{g}_i$ vs the base 10 logarithm of phylogenetic distance for each pair of strains in the synthetic community experiment (Fig. 4). The Pearson's correlation coefficient $\rho_{\text{phylo}} = -0.09$. Phylogenetic distance is calculated in a pairwise fashion by first aligning 16S rRNA gene sequences using the Biopython function pairwise2.align.globalms, scored with 1 point for identical characters, -1 point for non-identical characters, and -0.5 points for both opening and extending gaps. The alignment with the highest score was chosen, and phylogenetic distance $= 1 -$ matches/min(sequence length), where "matches" is the number of matching base pairs in the aligned sequences, and this value is normalized by the sequence length of the shorter sequence "min(sequence length)." **(B)** Statistical significance of ρ_{phylo} is assessed by resampling $n = 10000$ pairs of strains with replacement and re-calculating ρ_{phylo}. The resulting histogram of ρ_{phylo} values is shown. 95% of ρ_{phylo} values fall between -0.23 and 0.05, and the P value associated with a negative correlation, $P = 1 - \sum(\rho_{\text{phylo}} < 0)/n$, is 0.1. Black and red lines indicate the mean resampled ρ_{phylo} and the 95% confidence interval, respectively. There is therefore no statistically significant relationship between phylogenetic distance and growth rate vector in the synthetic community.
