# Supplementary material for: Timescale of environmental change modulates metabolic guild cohesion in microbial communities
Source: ISME J. 2025 Aug 22;19(1):wraf186. doi: 10.1093/ismejo/wraf186 (PMC12448442; doi:10.1093/ismejo/wraf186)
Supplement: SI_updated_refs_wraf186 [file si_updated_refs_wraf186.pdf]

Supplementary Information

Analytic approximation of the consumer covariance matrix

To derive the expected covariance matrix of strain abundances in slow fluctuation limit, we first rewrote Eq. 2 in matrix form as follows.

$$\frac{dX}{dt} = D(X)\left(G\frac{R}{R+R_0} - d_x\right) \tag{8}$$

$$\frac{dR}{dt} = K - D(d_R)R - D\left(\frac{R}{R+R_0}\right)C^T X \tag{9}$$

where R_0 is a constant affinity parameter, d_x is an N $\times$ 1 vector of consumer death rates d_R is an N $\times$ 1 vector of resource depletion rates, G is the growth rate matrix defined in the main text, where $G_{i,\alpha} = r_{i,\alpha}\gamma_{i,\alpha}$, C is the matrix comprised of $r_{i,\alpha}$, where $C_{i,\alpha} = r_{i,\alpha}$, K is the vector of resource supply rates, and D is used as the standard diag operator for vectors, where $D(X)$ refers to the diagonal matrix with the vector X on the diagonal.

The invertible case

For a given vector of resource supply rates, K, the equilibrium abundances of consumers, X^*, are given by:

$$X^* = \left(C^T\right)^{-1} D\left(\frac{R^*}{R^*+R_0}\right)^{-1}(K - D(d_R)R^*) \tag{10}$$

provided that the matrix C^T has a well-defined left inverse (or left pseudoinverse), which requires that the number of resources M is greater than or equal to N, the number of consumers.
In the special case where $M = N$, or the number of resources in the system is equal to the number of consumers, we can assume that the matrix G has a well-defined left inverse. In this case, using the consumer equations, we have that at equilibrium:

$$\frac{R^*}{R^*+R_0} = G^{-1}d_x \tag{11}$$

and

$$R^* = R_0\frac{G^{-1}d_x}{\mathbb{1} - G^{-1}d_x} \tag{12}$$

where $\mathbb{1}$ is the $M \times 1$ dimensional vector of all 1's.

Substituting these expressions into Eq. 10, we obtain:

$$X^* = \left(C^T\right)^{-1} \left(\frac{K}{G^{-1}d_x} - \frac{D(d_R)R_0}{\mathbb{1} - G^{-1}d_x}\right) \tag{13}$$

Then, we consider the response to a small environmental fluctuation in which $K \to K + \epsilon$. In this case, the new equilibrium abundance of consumers will be given by:

$$(X^*)' = \left(C^T\right)^{-1} \left(\frac{K+\epsilon}{G^{-1}d_x} - \frac{D(d_R)R_0}{\mathbb{1} - G^{-1}d_x}\right) \tag{14}$$

$$= X^* + \left(C^T\right)^{-1} \left(\frac{\epsilon}{G^{-1}d_x}\right) \tag{15}$$

Assuming the environmental fluctuations are given by an ensemble, $\rho(K)$, with a covariance matrix Σ_K, we can then write the expected covariance between consumers $\langle X_i, X_j\rangle$, as:

$$\langle X_i, X_j\rangle = \left(C^T\right)^{-1} \left(D(G^{-1}d_x)^{-1}\Sigma_K D(G^{-1}d_x)^{-1}\right) C^{-1}$$

(16)

In the limit of infinitely slow environmental fluctuations, a community is expected to equilibrate at an essentially constant influx rate K. Thus this expression, even though it was derived for discrete environmental perturbations, is the expected long-term covariance between consumers in the slow fluctuation limit.

Relationship between covariance matrix and overlap matrix

To relate the overlap matrix $O = GG^T$ to the covariance matrix, we first observe that each entry of the matrix C may be written as $C_{i,\alpha} = \frac{1}{\gamma_{i,\alpha}} G_{i,\alpha}$, where $\gamma_{i,\alpha}$ and $G_{i,\alpha}$ are given as above. If we assume that the yields take a constant value across all species and resources, or that $\gamma_{i,\alpha} = c \; \forall i, \alpha$ for some constant c, then the matrix C may be written as $C = \frac{1}{c}G$.

With this, and defining the matrix Z as:

$$Z = \left(D(G^{-1}d_x)^{-1}\Sigma_K D(G^{-1}d_x)^{-1}\right)^{-1}$$

We may then rewrite Eq. as:

$$\langle X_i, X_j\rangle = c^2 (GZG^T)^{-1} \tag{17}$$

Although the elements of the matrix Z will in general depend on the exact parameters of the sys-

tem, as well as the covariance matrix of environmental fluctuations Σ_K, it is nonetheless clear that the expected long-term covariance between consumers is defined by a scaled version of the matrix GG^T.

We consider a simplified scenario in which R^*_α, R_0 are identical for all resources R_α, and the matrix Σ_K is diagonal, implying that the covariance between any two influx rate fluctuations, K_i and K_j, is 0 for $i \neq j$.

In this case, the matrix $Z = dI$, a constant diagonal matrix, and Eq. may be further simplified as:

$$\langle X_i, X_j \rangle = \frac{c^2}{d}(GG^T)^{-1} \tag{18}$$

If the assumptions of this simplified scenario hold, we expect GG^T and $(GG^T)^{-1}$ to be good approximations for consumer covariances across short and long-timescale fluctuations, respectively. However, deviations from this simplified scenario, such as variance in the values of R^*_α for a given system, will mean that GG^T is likely to become an increasingly poor approximation of the covariance between consumers across all timescales.

The non-invertible case

The above expressions were derived under the assumption that the matrix G had a well-defined left-inverse, requiring that the number of consumers, N, is equal to the number of resources, M. However, this condition is not strictly necessary. Although the benefit of the preceding approach is that the expressions derived are expected to be exact in the case where there are no consumer extinctions, here we also employ a complementary approach to more thoroughly analyze the case in which we have arbitrary N, M.

We observe that at any equilibrium, the system defined by Eq. 9 must satisfy:

$$0 = G\frac{R^*}{R^* + R_0} - d_x \tag{19}$$

$$0 = K - D(d_R)R^* - D\left(\frac{R^*}{R^* + R_0}\right)C^T X^* \tag{20}$$

We again consider the system in response to perturbations to the vector of resource supply rates K, or perturbations in which $K \rightarrow K + \epsilon$. After such a perturbation to K, both consumer and resource equilibria will change, such that:

$$X^* \rightarrow X^* + \Delta X$$

$$R^* \to R^* + \Delta R$$

These perturbed equilibria must again satisfy the steady state equations defined by Eq. 20, such that:

$$0 = G\frac{R^* + \Delta R}{R^* + \Delta R + R_0} - d_x \tag{21}$$

$$K + \epsilon = D(d_R)(R^* + \Delta R) + D\left(\frac{R^* + \Delta R}{R^* + \Delta R + R_0}\right) C^T (X^* + \Delta X) \tag{22}$$

Although we cannot in general solve for ΔR and ΔX exactly, we can nonetheless estimate these quantities, the system's response to a perturbation in the resource supply vector, via a linear approximation. To do so, we expand around the original equilibrium point of the system to first order. Because we expand around an equilibrium, the zeroth order terms are 0. This yields the expression:

$$0 = GD\left(\frac{R_0}{(R^* + R_0)^2}\right)\Delta R \tag{23}$$

$$\epsilon = \left(D(d_R) + D\left(\frac{R_0}{(R^* + R_0)^2}\right) D(C^T X^*)\right)\Delta R + D\left(\frac{R}{R^* + R_0}\right) C^T \Delta X \tag{24}$$

If we collect these terms, we can rewrite Eq. 24 in matrix form to obtain the expression:

$$\begin{bmatrix} 0 & \hat{G} \\ \hat{C} & M \end{bmatrix} \begin{bmatrix} \Delta X \\ \Delta R \end{bmatrix} = \begin{bmatrix} 0 \\ \epsilon \end{bmatrix} \tag{25}$$

where $\hat{G} = GD\left(\frac{R_0}{(R^*+R_0)^2}\right)$, $M = \left(D(d_R) + D\left(\frac{R_0}{(R^*+R_0)^2}\right) D(C^T X)\right)$, and $\hat{C} = D\left(\frac{R^*}{R^*+R_0}\right) C^T$. We would like to solve for the perturbed strain abundances, ΔX. Manipulating Eq. 25, we obtain:

$$\begin{bmatrix} \Delta X \\ \Delta R \end{bmatrix} = \begin{bmatrix} 0 & \hat{G} \\ \hat{C} & M \end{bmatrix}^{-1} \begin{bmatrix} 0 \\ \epsilon \end{bmatrix} \tag{26}$$

We are then able to explicitly solve for ΔX using the standard formula for the inverse of 2×2 block matrices, yielding:

$$\Delta X = (\hat{G} M^{-1} \hat{C})^{-1} \hat{G} M^{-1} \epsilon \tag{27}$$

Substituting the above definitions of $\hat{G}$ and $\hat{C}$, and defining the matrix Z as $D\left(\frac{R_0}{(R^*+R_0)^2}\right) M^{-1} D\left(\frac{R}{R^*+R_0}\right)$, we can further simplify to obtain:

$$\Delta X = (GZC^T)^{-1}\hat{G}M^{-1}\epsilon \tag{28}$$

where this expression is analogous to that derived in Eq. 15 above. If the matrix G were taken to be left-invertible this approximation, obtained via a linear expansion around a fixed point, would reduce to the exact expression obtained in Eq. 15.

We are then able to employ Eq. 28 to approximate the expected covariance between consumers at long time scales in the same manner as above. If we again assume the environmental fluctuations are given by an ensemble, $\rho(K)$, with a covariance matrix Σ_K, we can then write the expected covariance between consumers $\langle X_i, X_j \rangle$, as:

$$\langle X_i, X_j \rangle = P\Sigma_K P^T \tag{29}$$

where the matrix P is taken to be $(GZC^T)^{-1}\hat{G}M^{-1}$. Analogously to the above case, we expect Eq. 29 to be a reasonable approximation of the consumer covariance matrix in the limit of slow environmental fluctuations (Fig. S1).

Deriving correlation matrices from covariance matrices

The strain-strain covariance matrices derived above (e.g., Eq. 29 or 17) can be converted to correlation matrices through standard normalization.

The correlation between strains i and j is calculated from the covariance matrix as:

$$R_{ij} = \frac{\langle X_i, X_j \rangle}{\sqrt{\langle X_i, X_i \rangle \langle X_j, X_j \rangle}} \tag{30}$$

where $\langle X_i, X_j \rangle$ is the covariance between strains i and j, normalized by the product of their standard deviations.

Cross-feeding interactions produce positive correlations on long timescales

Here, we consider cross-feeding interactions [1] in addition to resource competition interactions. Such interactions are common [2–5], and occur when one strain consumes a resource and subsequently excretes a different resource that can be consumed by another strain, such as in overflow metabolism [6]. We model these interactions here as follows.

$$
\begin{aligned}
\frac{dx_i}{dt} &= x_i\left(\sum_{\alpha=1}^{M} r_{i,\alpha}\gamma_{i,\alpha}(1-l_\alpha)\frac{R_\alpha}{R_\alpha+R_0} - d_x\right) \\
\frac{dR_\alpha}{dt} &= K_\alpha(t) + \sum_{i=1}^{N}\left[-r_{i,\alpha}\frac{R_\alpha}{R_\alpha+R_0}x_i + \sum_{\beta=1}^{M} r_{i,\beta}\frac{R_\beta}{R_\beta+R_0}x_i l_\beta T_{\alpha,\beta}\right] - R_\alpha d_R
\end{aligned}
\tag{31}
$$

where l_α are the leakage coefficients encoding the fractions of resources α that are excreted, and $T_{\alpha,\beta}$ are binary cross-feeding coefficients determining whether resource β is converted to resource α. In the low resource limit, $R_\alpha \ll R_0$, Eq. 31 reduces to a commonly used form in microbial ecology [7].

To test the effect of cross-feeding on community response to environmental fluctuations, we numerically integrate Eq. 31 with the sinusoidal resource influx rates of Eq. 3. Our first cross-feeding structure simulates trophic cross-feeding, where the orange guild consumes primary resources and supplies them to the blue guild. In particular, strains in the orange guild excrete resources 41-80 after consuming resources 1-40 ($l_{\alpha\leq 40} = 0.5$ and $T_{\alpha=\beta+40,\beta\leq 40} = 1$; Fig. S7B). This results in cross-feeding interactions where the orange guild supplies resources to the blue guild (Fig. S7C). We also set $K_{40<\alpha\leq 80}(t) = 0$, so that the only block resources available to the blue guild are supplied by the orange guild. In this cross-feeding case, we see intra-guild cohesion on short timescales but not on long timescales (correlated dynamics, Fig. S7C). This matches the non-cross-feeding case qualitatively. *Inter*-guild dynamics, however, are correlated across all timescales due to the facilitation of blue guild growth by the orange guild, with these values decreasing to small but positive correlations at long timescales (Fig. S7C, right panels). This is distinct from the resource competition case, where we do not observe such inter-guild correlations (Fig. 2).

The second cross-feeding structure mimics specific cross-feeding, where each strain in the orange guild excretes the private resource of a strain in the blue guild after consuming its own private resource ($l_{41\leq\alpha\leq 50} = 0.5$ and $T_{\alpha=\beta+10,\beta>50} = 1$; Fig. S7E). We also set $K_{\alpha>45}(t) = 0$ and omit the blue block resources, so that the only private resources available to the blue guild were supplied by the orange guild (Fig. S7D). In this case, as in previous cases, we see a cohesive intra-guild response on short, but not long, timescales (Fig. S7G). Strong positive correlations between cross-feeding partners across guilds (strains 1 and 6, 2 and 7, 3 and 8, and so on) are observed on all timescales (Fig. S7F). Overall, these results show that intra-guild cohesion is robust to qualitatively different inter-guild interaction mechanisms. Furthermore, the correlations between strains interacting via cross-feeding appear to be correlated on all timescales, with the strength of the correlation increasing with the specificity of the interaction. This is qualitively distinct from strains

interacting via resource competition, which are correlated on short timescales and anti-correlated on long timescales. This observation suggests that inference of interaction type may be made by studying correlation functions between strains subject to differing environmental fluctuation rates.

Interpretation and effect of private resources

In this study, we provide each strain with a fluctuating "private resource" that ensures that it maintains some significant biomass (Fig. 1A). This ensures that the community remains relatively stable over time, which is generally the case in naturally occuring communities [8–11].
Ecologically, such private resources need not be interpreted literally as nutrients available only to a single member of the community. Instead, these resources may be viewed more abstractly as some mechanism that is orthogonal to the block resources that maintains diversity within metabolic guilds, such as a spatial structure or phage predation [12–15]. For instance, the background fluctuations introduced by the private resources may arise from migration from different environments or adherence to surfaces that provide unique access to resources [16, 17].
Although it is in principle possible for block resources alone to support coexistence, identifying a community that coexists across an ensemble of fluctuating resources requires significant fine-tuning. In particular, we expect that even *in silico* communities chosen such that members coexist at average values of the block resources will experience extinctions in fluctuating environments. Although it is possible that such fine-tuning will arise from ecological and evolutionary processes, it necessarily biases the community composition and implicitly makes an assumption about the source of community stability. For this reason, we chose to maintain coexistence using private resources, allowing us to choose an unbiased distribution of traits within metabolic guilds. Although extensive theoretical progress has been made regarding the source of community stability and strain coexistence [18–20], the effect of such processes on community cohesion dynamics has not been considered. This remains an interesting avenue for future research.

References

[1] D'Souza G, Shitut S, Preussger D, Yousif G, Waschina S, Kost C. Ecology and evolution of metabolic cross-feeding interactions in bacteria. Nat Prod Rep. 2018;35(5):455-88.

[2] Evans CR, Kempes CP, Price-Whelan A, Dietrich LE. Metabolic heterogeneity and cross-feeding in bacterial multicellular systems. Trends Microbiol. 2020;28(9):732-43.

[3] Goldford JE, Lu N, Bajić D, Estrela S, Tikhonov M, Sanchez-Gorostiaga A, et al. Emergent simplicity in microbial community assembly. Science. 2018;361(6401):469-74.

[4] Crocker K, Lee KK, Chakraverti-Wuerthwein M, Li Z, Tikhonov M, Mani M, et al. Environmentally dependent interactions shape patterns in gene content across natural microbiomes. Nat Microbiol. 2024;9(8):2022-37.

[5] Gowda K, Ping D, Mani M, Kuehn S. Genomic structure predicts metabolite dynamics in microbial communities. Cell. 2022;185(3):530-46.

[6] Basan M, Hui S, Okano H, Zhang Z, Shen Y, Williamson JR, et al. Overflow metabolism in *Escherichia coli* results from efficient proteome allocation. Nature. 2015;528(7580):99-104.

[7] Marsland III R, Cui W, Mehta P. The minimum environmental perturbation principle: A new perspective on niche theory. Am Nat. 2020;196(3):291-305.

[8] Martin-Platero AM, Cleary B, Kauffman K, Preheim SP, McGillicuddy DJ, Alm EJ, et al. High resolution time series reveals cohesive but short-lived communities in coastal plankton. Nat Commun. 2018;9(1):266.

[9] Gibbons SM, Kearney SM, Smillie CS, Alm EJ. Two dynamic regimes in the human gut microbiome. PLoS Comput Biol. 2017;13(2):e1005364.

[10] Relman DA. The human microbiome: ecosystem resilience and health. Nutr Rev. 2012;70(suppl_1):S2-9.

[11] Faust K, Lahti L, Gonze D, De Vos WM, Raes J. Metagenomics meets time series analysis: unraveling microbial community dynamics. Curr Opin Microbiol. 2015;25:56-66.

[12] Mattei M, Arenas A. Exploring spatial segregation induced by competition avoidance as driving mechanism for emergent coexistence in microbial communities. Phys Rev E. 2024;110(1):014404.

[13] Gude S, Pinçe E, Taute KM, Seinen AB, Shimizu TS, Tans SJ. Bacterial coexistence driven by motility and spatial competition. Nature. 2020;578(7796):588-92.

[14] Simmons EL, Bond MC, Koskella B, Drescher K, Bucci V, Nadell CD. Biofilm structure promotes coexistence of phage-resistant and phage-susceptible bacteria. mSystems. 2020;5(3):10-1128.

[15] Scanlan PD. Bacteria–bacteriophage coevolution in the human gut: implications for microbial diversity and functionality. Trends Microbiol. 2017;25(8):614-23.

[16] Chen X, Wang M, Luo L, An L, Liu X, Fang Y, et al. High immigration rates critical for establishing emigration-driven diversity in microbial communities. Cell Syst. 2024;15(3):275-85.

[17] Dang H, Lovell CR. Microbial surface colonization and biofilm development in marine environments. Microbiol Mol Biol Rev. 2016;80(1):91-138.

[18] Pearce MT, Agarwala A, Fisher DS. Stabilization of extensive fine-scale diversity by ecologically driven spatiotemporal chaos. Proc Natl Acad Sci USA. 2020;117(25):14572-83.

[19] Mahadevan A, Fisher DS. Continual evolution in nonreciprocal ecological models. arXiv. 2024. https://doi.org/10.48550/arXiv.2411.17148.

[20] Narla AV, Hwa T, Murugan A. Dynamic coexistence driven by physiological transitions in microbial communities. Proc Natl Acad Sci USA. 2025;122(16):e2405527122.
