# Supplementary material for: Timescale of environmental change modulates metabolic guild cohesion in microbial communities
Source: ISME J. 2025 Aug 22;19(1):wraf186. doi: 10.1093/ismejo/wraf186 (PMC12448442; doi:10.1093/ismejo/wraf186)
Supplement: Figure_S7_wraf186 [file figure_s7_wraf186.pdf]

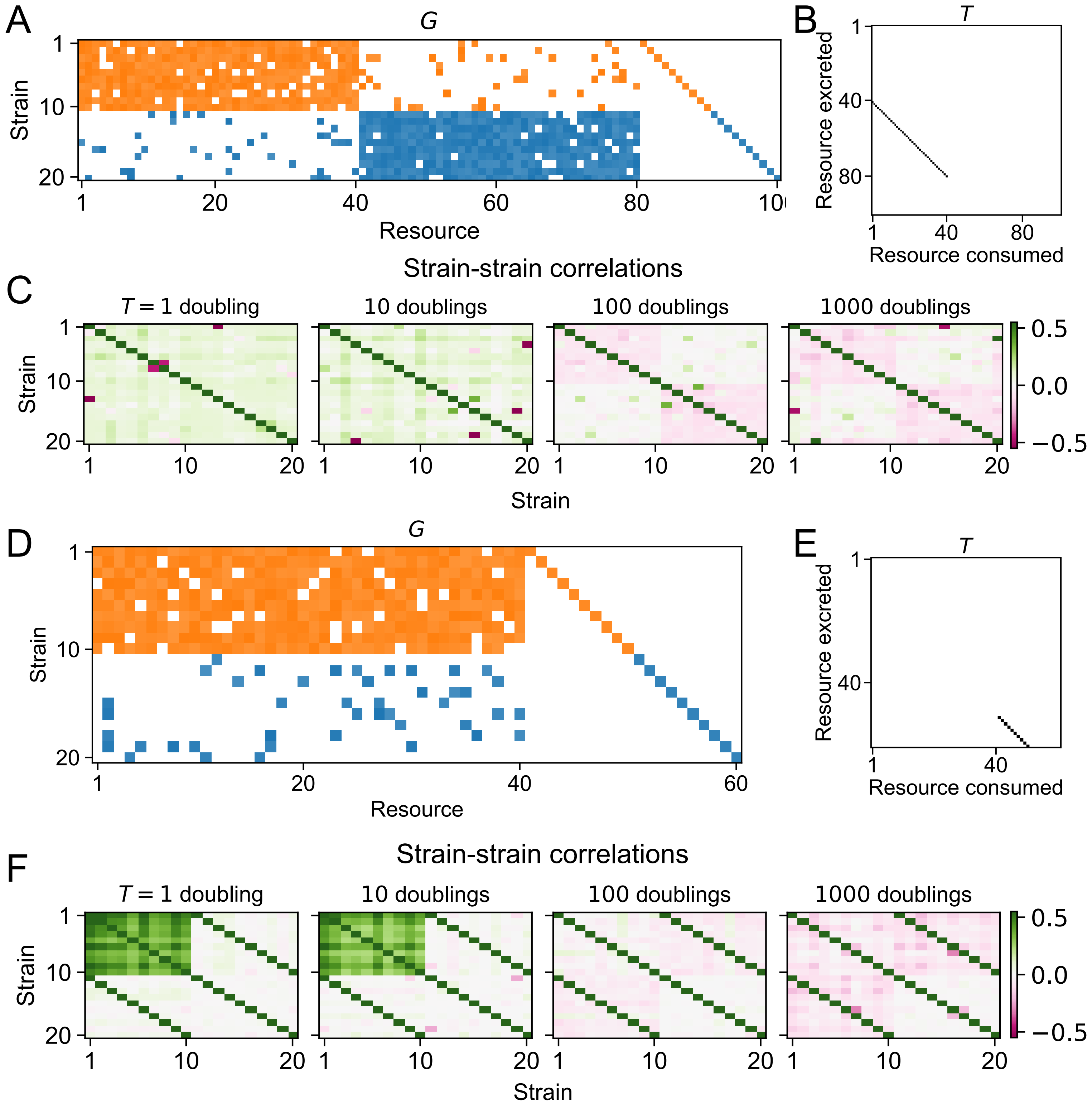
A
G
Strain
Resource
B
T
Resource excreted
Resource consumed
Strain-strain correlations
C
T = 1 doubling
10 doublings
100 doublings
1000 doublings
Strain
D
G
Strain
Resource
E
T
Resource excreted
Resource consumed
Strain-strain correlations
F
T = 1 doubling
10 doublings
100 doublings
1000 doublings
Strain
